# Paneth cells as the cornerstones of intestinal and organismal health: a primer

**DOI:** 10.15252/emmm.202216427

**Published:** 2022-12-27

**Authors:** Charlotte Wallaeys, Natalia Garcia‐Gonzalez, Claude Libert

**Affiliations:** ^1^ Center for Inflammation Research‐VIB Ghent Belgium; ^2^ Department of Biomedical Molecular Biology Ghent University Ghent Belgium

**Keywords:** paneth cells, gut homeostasis, infection, antimicrobial peptides, Digestive System

## Abstract

Paneth cells are versatile secretory cells located in the crypts of Lieberkühn of the small intestine. In normal conditions, they function as the cornerstones of intestinal health by preserving homeostasis. They perform this function by providing niche factors to the intestinal stem cell compartment, regulating the composition of the microbiome through the production and secretion of antimicrobial peptides, performing phagocytosis and efferocytosis, taking up heavy metals, and preserving barrier integrity. Disturbances in one or more of these functions can lead to intestinal as well as systemic inflammatory and infectious diseases. This review discusses the multiple functions of Paneth cells, and the mechanisms and consequences of Paneth cell dysfunction. It also provides an overview of the tools available for studying Paneth cells.

## Graphical abstract

This Review discusses biological functions of Paneth cells and their importance for intestinal homeostasis and organismal health.

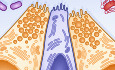

**Glossary**
PCs: Paneth cells are secretory cells located in the crypts of Lieberkühn, adjacent to the intestinal stem cells. They produce antimicrobial peptides and proteins and other components that are important in host defense and immunity.
α‐defensins: Enteric α‐defensins are antimicrobial peptides stored in the secretory granules of PCs. They are responsible for most of the antimicrobial activity of PCs.
LYZ: Lysozyme is the first antimicrobial peptide discovered in PCs and is widely used as a PC marker in the small intestine.
Wnt/β‐catenin signaling pathway: Wingless‐related integration site (Wnt)/β‐catenin signaling pathway is a signal transduction pathway regulating intestinal stem cell self‐renewal and differentiation. The Wnt/β‐catenin pathway is most active at the intestinal crypt base.
Notch signaling: Notch signaling in the gut directs the differentiation of progenitor cells into absorptive cells (enterocytes) by inhibiting secretory cell differentiation (Goblet cells, enteroendocrine cells, tuft cells, and PCs) *via* the expression of the transcription factor Hairy and enhancer of split 1 (HES1).
miRNA: microRNA is a small single‐stranded non‐coding RNA molecule that regulates gene expression on a post‐transcriptional level.
mTORC1: Mammalian target of rapamycin complex 1 is a serine/threonine protein kinase and serves as a sensor for a wide range of environmental factors, for example, nutrient availability. The protein responds to environmental triggers by adapting transcription, translation and autophagy, and can thereby regulate several cellular processes.
Autophagy: Autophagy is a process by which a cell removes damaged or unnecessary components via lysosome‐dependent degradation. It is a fundamental cell survival mechanism contributing to the mobilization of cellular energy stores and is thus critical for maintaining the homeostasis of cells.
Endoplasmic reticulum: Endoplasmic reticulum is an intracellular organelle that plays a key role in, for example, folding, modifying, and sorting newly synthesized proteins and the synthesis of cellular lipids.
ER stress: Endoplasmic reticulum stress is induced by the accumulation of unfolded or misfolded proteins. Cells activate signaling pathways (unfolded protein response; UPR) to deal with ER stress.
Mucus: The mucus layer in the intestinal epithelium forms a physical and immunological barrier to protect the epithelium from infiltration of microorganisms and other components.

## Introduction and definition of Paneth cells

Paneth cells (PCs) are found in the crypts of the small intestine of many mammals (Lueschow & McElroy, 2020). They are highly secretory cells with a lifespan of about 2 months (Ireland *et al*, 2005). Their secretory function is hallmarked by a pyramidal‐shaped morphology, abundant endoplasmic reticulum (ER), well‐developed Golgi network and apically oriented secretory granules. These granules accumulate antimicrobial peptides and proteins (AMPs), enzymes and growth factors, many of which are crucial in host defense (Ayabe *et al*, 2000). Hence, PCs might control the composition of the enteric microbiota and maintain gut homeostasis. Alterations in PCs or PC dysfunction contribute to several diseases, for example, graft‐versus‐host disease (GVHD) and inflammatory bowel disease (IBD), including Crohn's disease (CD) and ulcerative colitis (Levine *et al*, 2013; Deuring *et al*, 2014). Understanding PCs and expanding the tools to study them is therefore of utmost importance. This review focusses on their biological functions, how they can be modulated by environmental conditions and disease, and the tools available to study them.

## PC ontogeny

The enteric epithelium consists of a monolayer of columnar cells with diverse functions and plays a crucial role in metabolism, preserving intestinal homeostasis, and defense of the body (Ali *et al*, 2020). Its architecture is composed of upright villi interspersed by the crypts of Lieberkühn. The epithelium of the small intestine consists of five main cell types differentiated from Lgr5+ intestinal stem cells (ISCs): enterocytes, goblet cells, enteroendocrine cells, tuft cells, and PCs (Van Der Flier & Clevers, 2009). Due to the daily challenge of food components and bacteria, the epithelium is constantly renewed to maintain homeostasis and preserve the integrity of the intestinal lining (Barker *et al*, 2012). ISCs are responsible for the rapid renewal and replenishment of the epithelium by forming progenitor cells that can differentiate further. The major part of differentiated cells moves upwards in the villus, with a lifespan of 3–5 days, and are then shed into the lumen at the top of the villus, where they die by a process called anoikis (Cheng & Leblond, 1974). However, PCs move downwards into the crypts of Lieberkühn, where they exert versatile functions, including ISC support. In some mammals, such as humans and mice, PCs are abundant and easy to observe, but in other animals (e.g., pigs), their existence is controversial (Myer, 1982; van der Hee *et al*, 2018).

### PC formation and differentiation

**Figure 1:**
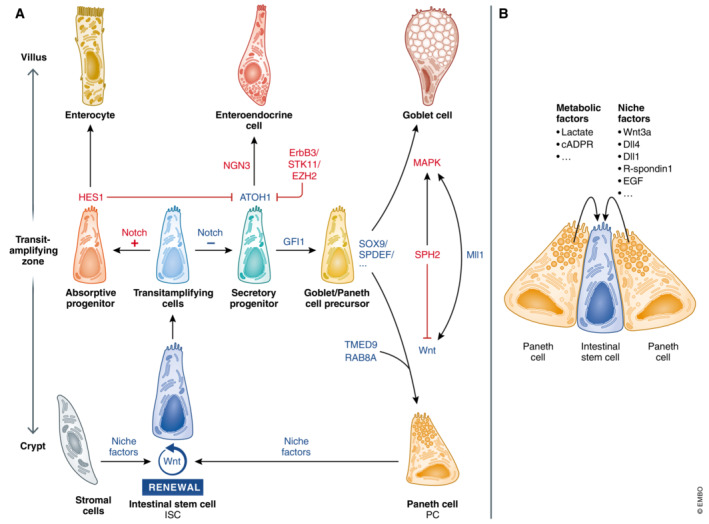
Intestinal cell differentiation pathways and signals (A) Wnt and Notch signals control ISC renewal and differentiation. PCs and for example, pericryptal stromal cells support the stemness of ISCs by providing niche factors. ISCs in the lower part of the crypt give rise to a larger pool of transit‐amplifying cells in the transit‐amplifying zone. Differentiation from transit‐amplifying cells towards secretory or absorptive progenitors is initially controlled by Notch signaling. Cells that receive Notch signals express HES1, a negative regulator of ATOH1, leading to differentiation into absorbing enterocytes. Cells devoid of Notch express ATOH1, a hallmark of secretory cells. GFI1 acts downstream of ATOH1 to select for goblet cell and PC differentiation, as it represses neurogenin 3 (NGN3), a transcription factor involved in enteroendocrine cell differentiation. ErbB3, STK11, and EZH2 influence PC differentiation by reducing ATOH1 levels and affect the numbers and/or location of mature PCs. SOX9 and SPDEF are involved in PC and goblet cell differentiation. Reduced MAPK signaling and increased Wnt signaling (influenced by Wnt regulating factors, e.g., RAB8A, TMED9, SPH2, and Mll1) favor differentiation into PCs instead of goblet cells. (B) PCs support ISCs by providing niche and metabolic factors. Blue – factors that promote PC differentiation, red – factors that inhibit PC differentiation.

ISCs in the lower part of the crypts give rise to a large pool of transit‐amplifying cells. The most important regulators of ISC activity are Wnt, bone morphogenetic protein (BMP), Notch, and epidermal growth factor (EGF) signaling pathways. These factors are gradually expressed along the villi and crypts (Malijauskaite *et al*, 2021). Transit‐amplifying cells remain 2 days in the transit‐amplifying zone (higher part of the crypts), where they multiply and mature into differentiated intestinal epithelial cells (IECs, Fig 1) (Van Der Flier & Clevers, 2009).

The PC differentiation is initially controlled by Notch signaling. Progenitor cells that are deficient in Notch receptor express atonal BHLH transcription factor 1 (ATOH1), the essential driver of secretory cell differentiation (Yang *et al*, 2001). Progenitor cells with high Notch activity induce HES1, a negative regulator of ATOH1, making them prone to differentiate into absorbing enterocytes. Growth factor independent 1 transcriptional repressor (GFI1) acts downstream of ATOH1 to select for differentiation of goblet cells and PCs, as it transcriptionally represses differentiation towards enteroendocrine cells by repressing the transcription factor neurogenin 3 (Bjerknes & Cheng, 2010).

Thus, factors that affect ATOH1 influence the differentiation towards PCs (and other secretory cells). PKC_λ/**Ι**_ is such a factor, as it destabilizes EZH2, an ATOH1 suppressor co‐localized with PC markers in the crypts. PKC_λ/**Ι**_^ΔIEC^ mice (lacking *Prkci*, which encodes PKC_λ/**Ι**_, specifically in the intestinal epithelium) had reduced ATOH1 and GFI1 crypt levels, and less mature PCs, along with an increase in an intermediate cell‐type positive for both PC and goblet cell markers (Nakanishi *et al*, 2016). Erb‐b2 receptor tyrosine kinase 3 (ErbB3) and serine/threonine kinase 11 (STK11) block ATOH1 *via* the PI3K–Akt pathway and PDK4, respectively, suppressing PC differentiation. Interestingly, ErbB3 knockout (KO) mice (*ErbB3* ^KO^) had increased lysozyme (LYZ, encoded in human by *LYZ*, and in mice by *Lyz1*) expressing PCs but no change in other secretory cells (Almohazey *et al*, 2017).

ATOH1/GFI1‐positive precursor cells can further differentiate towards PCs, a process that is not fully understood but strongly depends on (1) transcription factors and downstream effectors of Wnt/β‐catenin signaling and (2) factors that can influence Wnt/β‐catenin signaling (van Es *et al*, 2005; Van Der Flier & Clevers, 2009; Grinat *et al*, 2022).

1. Active Wnt signaling promotes PC differentiation. Wnt activation causes stabilization and translocation of β‐catenin to the nucleus, where it interacts with several T cell factor (TCF) molecules to induce a unique differentiation profile. TCF4 is a regulator of PC maturation and induces a PC gene program in the embryonic intestine of mice (van Es *et al*, 2005). SRY‐Box transcription factor 9 (SOX9) and SAM‐pointed domain containing ETS transcription factor (SPDEF) are involved in PC and goblet cells differentiation (Bastide *et al*, 2007; Gregorieff *et al*, 2009). The unique positioning of PCs towards the crypts is tightly regulated by the Wnt target ephrin receptor B3 (EphB3). Pathways downstream of ATOH1 often affect both goblet cells and PCs, and further research is needed to distinguish their differentiation. However, it is known that persistently active Wnt signaling in the secretory progenitors favors PC differentiation (van Es *et al*, 2005).
2. Spherocytosis 2 protein (SPH2), ras‐related protein (RAB8A) and Krüppel‐like factor 5 (KLF5) can influence Wnt/β‐catenin signaling. Ablation of SPH2 reduces ERK1/2‐MAPK signaling, corresponding with higher Wnt β‐catenin/TCF4 signaling and differentiation towards PCs (Heuberger *et al*, 2014). Ablation of RAB8A, a protein involved in Wnt secretion, reduces Wnt signaling and PC number (Das *et al*, 2015). Deletion of the transcription factor *Klf5* reduces PC and goblet cell numbers (Nandan *et al*, 2014). Also, miRNAs can influence PC function and differentiation; for example, miRNA‐802 represses TMED9, a stimulator of Wnt and LYZ/α‐defensins secretion in PCs (Goga *et al*, 2021). Besides, the epigenetic factor Mll1 can influence the differentiation towards secretory cells in a dual way. Mll1 is involved in keeping stemness and preventing differentiation but also has a role in determining PC and goblet cell fate by coordinating Wnt and MAPK signaling in the progenitor cells. *Mll1* ^KO^ mice had increased secretory cells, but impaired PCs and goblet cell specification, as these cells were positive for both PC and goblet cell markers (Grinat *et al*, 2022).

Paneth cell differentiation is a complex interplay between different factors. Reduced Notch, strong Wnt, and weak MAPK signaling in progenitor cells promote PC differentiation. Notably, most research on PC differentiation and ISC niche has been performed in mice, but there can be differences between species, for example, there are important differences in niche factors needed in human and mouse organoids (Sato *et al*, 2011b).

### PC numbers

Paneth cells increase in the proximal to distal direction of the small intestine, which corresponds with increased bactericidal activity (Nakamura *et al*, 2020). The number of PCs per crypt in mice vary between strains and the techniques used but are estimated at 5–16 PCs per crypt (Nakamura *et al*, 2020). Inbred strains under identical housing conditions differ in the number of PCs and the expression of AMPs. For example, 129/SvEv mice have fewer PCs than C57BL/6J mice (Gulati *et al*, 2012). This identifies a critical role of the host genotype in PC activity and the intestinal microbiota. Such differences might be of interest to search for modifier genes that influence PC numbers and activities, but such inbred strains have been established as homozygous lines and kept in captivity over many decades. This environmental pressure may have led to genetic drifts that have little relevance to natural environments. PC studies on wild mice and wildling mice might be more relevant (but also more complex) than inbred lines kept in captivity.

## PC functions in physiology and homeostasis

The best‐known function of PCs is controlling the microbiome composition, but they have much broader functions. PCs can support and communicate with ISCs, they are involved in metal uptake, and can perform phagocytosis, efferocytosis and preserve barrier function.

### PCs and the ISC niche: support and communication

The unique crypt morphology of terminally differentiated PCs interspersed between pluripotent ISCs indicates the interactivity of these two crypt cells. The study of developmental issues in villus–crypt proliferation and differentiation led to insights into ISC maintenance and plasticity. One insight is that PCs produce essential niche signals for ISCs: Wnt family member 3A (Wnt3a), EGF, R‐spondin 1, Notch ligands (Dll4 and Dll1), and transforming growth factor α (TGFα) (Sato *et al*, 2011a). PCs' support of ISC stemness, and ISCs' differentiation into PCs illustrate their strong interdependence (Mei *et al*, 2020). *In vivo* PC ablation in three genetic mouse models (CR2‐tox176 mice, *Gfi1* mutation, and conditional deletion of *Sox9*) leads to similar losses in ISCs (Sato *et al*, 2011a). The dependency of ISC stemness on PCs has also been shown *in vitro*, as the absence of PCs largely prevents organoid formation from ISCs. However, the effect on ISCs seems to depend on the PC‐ablation model: loss of *Atoh1* in the gut induced PC ablation but did not affect the number of ISCs (Durand *et al*, 2012). Some studies indicated a role for pericryptal stromal cells in providing niche factors (Durand *et al*, 2012; McCarthy *et al*, 2020). Moreover, PC ablation by using a diphtheria toxin receptor gene inserted into the *Lyz1* locus led to the appearance of alternative niche cells (enteroendocrine and tuft cells) that provided niche factors to ISCs (Van Es *et al*, 2019).

Certain bacterial communities (such as *Bifidobacterium* spp. and *Lactobacillus* spp.) can communicate with ISCs *via* PCs (Lee *et al*, 2018). They produce lactic acid in the lumen, which binds to the recently discovered lactate G‐protein‐coupled receptor (Gpr81, encoded by *Hcar1*) on PCs. This interaction triggers PCs to increase *Wnt3a* expression and then stimulates Wnt signaling in ISCs (Lee *et al*, 2018). PCs can also rely on their glycolytic metabolism to produce lactate, which is then excreted and passed to ISCs. This lactate is oxidized by ISCs to pyruvate and serves as a fuel for the TCA cycle to generate ATP and reactive oxygen species (ROS). The latter is required to keep up high levels of phosphorylated P38, a MAP kinase important for regulation of ISCs self‐renewal, differentiation, and crypt formation (Rodríguez‐Colman *et al*, 2017). PCs can also serve as sensors of the nutritional status of the organism and communicate it to adjacent ISCs *via* mTORC1. Caloric restriction attenuates mTORC1 signaling in PCs, followed by increased bone stromal antigen (Bst1) levels. Bst1 is an ectoenzyme that converts NAD+ to cyclic ADP ribose, a paracrine product that promotes ISC self‐renewal, while reducing the pool of more differentiated progenitor cells (Yilmaz *et al*, 2012). Moreover, in pathological conditions, PCs can de‐differentiate into ISCs (Schmitt *et al*, 2018). PC–ISC interactions have been reviewed (Mei *et al*, 2020) and will not be discussed much in this review.

### Controlling microbiome composition

Paneth cells are the main producers of AMPs in the gastrointestinal tract, making them key players in sensing and controlling the microbiome composition. Thereby, they can preserve homeostasis and prevent bacteria from crossing the intestinal barrier. PCs produce a unique repertoire of AMPs in the gut, for example, LYZ, α‐defensins (called cryptdins in mice) and cryptdin‐related sequence peptides. AMPs are stored in secretory granules and released at the apex of PCs into the crypt lumen. In addition, PCs also produce AMPs that are not restricted to PCs in the gastrointestinal tract, for example, Regenerating islet‐derived protein 3 gamma (Reg3γ), secretory phospholipase A2 IIA (sPLA_2_ IIA), and Angiogenin‐4. A review elegantly describes the general intestinal AMPs in detail (Bevins & Salzman, 2011), so this review will focus on the AMPs that are exclusively produced by PCs in the gut.

#### Action mechanism of PC‐specific AMPs in the gut

LYZ was the first AMP discovered in PCs and is widely used as a PC marker in the ileum (Haber *et al*, 2018). The most abundant AMPs in PCs are α‐defensins, which are also produced by some myeloid‐derived cells (Selsted & Ouellette, 1995). The structures, action mechanisms, and functions of both AMPs are listed in Table 1. The cryptdin‐related sequence peptides share similarities with α‐defensin in their prosegment but the amount and positioning of the cysteines in the mature part are different.

**Table 1:** The structure, mechanisms, and functions of lysozyme and α‐defensins.

Lysozyme	Structure and antimicrobial activity	β‐1,4‐N‐acetylmuramoylhydrolase: Glycosidase responsible for enzymatic hydrolysis of peptidoglycans. This causes instability in the cell wall particularly of Gram‐positive bacteria	Ragland & Criss (2017)
		Cationic protein: The cationic structure leads to electrostatic interaction with phospholipids in the bacterial membrane. This results in the formation of pores in the membranes of both Gram‐positive and Gram‐negative bacteria, and subsequent bacterial death	Derde *et al* (2013), Ragland & Criss (2017)
	Other activities	It also has antiviral, antineoplastic and as antioxidant properties	Sava *et al* (1989), Croguennec *et al* (2000), Małaczewska *et al* (2019)
α‐Defensins	Structure and antimicrobial activity	Mature peptide consisting of six conserved cysteines forming three intramolecular disulfide bonds stabilizing a β‐sheet structure	Selsted & Ouellette (1995)
		The mature peptide is cationic and amphiphilic, leading to electrostatic interaction with phospholipids in the bacterial membrane. This results in the formation of membrane pores in both Gram‐positive and Gram‐negative bacteria, and subsequent bacterial death	Hadjicharalambous *et al* (2005)
	Other activities	It also has antiviral, antifungal and antiprotozoal activities	Daher *et al* (1986), Aley *et al* (1994), Kai‐Larsen *et al* (2007)

In mice, the MGI Genome Browser reports 43 annotated α‐defensin genes, nine cryptdin‐related sequence genes, and 18 α‐defensin pseudogenes (Table 2). Genetic differences in α‐defensins exist among mouse strains, which can affect PC studies in specific mouse backgrounds. Despite the many annotated genes in mice, not all are found at the protein level (Shanahan *et al*, 2011). The human genome encodes 10 α‐defensins but produces only two enteric α‐defensins (HD5 and HD6) (Patil *et al*, 2005). However, humans express diverse neutrophilic α‐defensins, which is not the case in mice (Shanahan *et al*, 2011). Human HD6 differs from other α‐defensins (e.g., cryptdins and HD5) by having an extra antimicrobial function, that is, the formation of self‐assembled peptide nano‐nets to capture bacteria (Chu *et al*, 2012; Schroeder *et al*, 2015).

**Table 2:** Annotated α‐defensin genes, cryptdin‐related sequence genes, and α‐defensin pseudogenes in the MGI Genome Browser.

Name	MGI ID	Symbol
defensin, alpha 1	MGI:94880	Defa1
defensin, alpha, 2	MGI:94882	Defa2
defensin, alpha, 3	MGI:94883	Defa3
defensin, alpha, 4	MGI:99584	Defa4
defensin, alpha, 5	MGI:99583	Defa5
defensin, alpha, 6	MGI:99582	Defa6
defensin, alpha, 7	MGI:99581	Defa7
defensin, alpha, 8	MGI:99580	Defa8
defensin, alpha, 9	MGI:99579	Defa9
defensin, alpha, 10	MGI:99591	Defa10
defensin, alpha, 11	MGI:99590	Defa11
defensin, alpha, 12	MGI:99589	Defa12
defensin, alpha, 13	MGI:99588	Defa13
defensin, alpha, 14	MGI:99587	Defa14
defensin, alpha, 15	MGI:99586	Defa15
defensin, alpha, 16	MGI:99585	Defa16
defensin, alpha, 17	MGI:1345152	Defa17
defensin, alpha, 20	MGI:1915259	Defa20
defensin, alpha, 21	MGI:1913548	Defa21
defensin, alpha, 22	MGI:3639039	Defa22
defensin, alpha, 23	MGI:3630381	Defa23
defensin, alpha, 24	MGI:3630383	Defa24
defensin, alpha, 25	MGI:3630385	Defa25
defensin, alpha, 26	MGI:3630390	Defa26
defensin, alpha, 27	MGI:3642780	Defa27
defensin, alpha, 28	MGI:3646688	Defa28
defensin, alpha, 29	MGI:94881	Defa29, Defa‐rs1
defensin, alpha, 30	MGI:3808881	Defa30
defensin, alpha, 31	MGI:102509	Defa31Defa‐rs7
defensin, alpha, 32	MGI:3709042	Defa32
defensin, alpha, 33	MGI:5434357	Defa33
defensin, alpha, 34	MGI:3709048	Defa34
defensin, alpha, 35	MGI:3711900	Defa35
defensin, alpha, 36	MGI:5434853	Defa36
defensin, alpha, 37	MGI:3705236	Defa37
defensin, alpha, 38	MGI:3709605	Defa38
defensin, alpha, 39	MGI:3611585	Defa39
defensin, alpha, 40	MGI:3708769	Defa40
defensin, alpha, 41	MGI:3705230	Defa41
defensin, alpha, 42	MGI:3645033	Defa42
defensin, alpha, 43	MGI:3648003	Defa43
defensin, alpha, pseudogene 1	MGI:3630392	Defa‐ps1
defensin, alpha, pseudogene 2	MGI:3832603	Defa‐ps2
defensin, alpha, pseudogene 3	MGI:3705791	Defa‐ps3
defensin, alpha, pseudogene 4	MGI:3705782	Defa‐ps4
defensin, alpha, pseudogene 5	MGI:3705778	Defa‐ps5
defensin, alpha, pseudogene 6	MGI:3705855	Defa‐ps6
defensin, alpha, pseudogene 7	MGI:3705773	Defa‐ps7
defensin, alpha, pseudogene 8	MGI:3832672	Defa‐ps8
defensin, alpha, pseudogene 9	MGI:3705785	Defa‐ps9
defensin, alpha, pseudogene 10	MGI:3705783	Defa‐ps10
defensin, alpha, pseudogene 11	MGI:3705879	Defa‐ps11
defensin, alpha, pseudogene 12	MGI:3647175	Defa‐ps12
defensin, alpha, pseudogene 13	MGI:3705864	Defa‐ps13
defensin, alpha, pseudogene 14	MGI:3705817	Defa‐ps14
defensin, alpha, pseudogene 15	MGI:3705808	Defa‐ps15
defensin, alpha, pseudogene 16	MGI:3705774	Defa‐ps16
defensin, alpha, pseudogene 17	MGI:3705788	Defa‐ps17
defensin, alpha, pseudogene 18	MGI:3642785	Defa‐ps18
defensin, alpha, related sequence 2	MGI:99592	Defa‐rs2
defensin, alpha, related sequence 4	MGI:102512	Defa‐rs4
defensin, alpha, related sequence 5	MGI:102511	Defa‐rs5
defensin, alpha, related sequence 6	MGI:102510	Defa‐rs6
defensin, alpha, related sequence 8	MGI:102508	Defa‐rs8
defensin, alpha, related sequence 9	MGI:102507	Defa‐rs9
defensin, alpha, related sequence 10	MGI:102516	Defa‐rs10
defensin, alpha, related sequence 11	MGI:102515	Defa‐rs11
defensin, alpha, related sequence 12	MGI:102514	Defa‐rs12

The α‐defensins are produced as pre‐pro‐peptides. First, they lose their signal peptide while moving from the ER into the secretory vesicles. Then, proteolytic cleavage turns the pro‐defensin into an active α‐defensin. In humans, this proteolytic maturation is executed by trypsin, which is stored as a proenzyme (trypsinogen) in the PCs and activated after or during secretion (Ghosh *et al*, 2002). In mice, proteolytic maturation is performed by matrix metalloproteinase 7 (MMP7, also known as matrilysin) (Wielockx *et al*, 2004).

Wilson *et al* (1999) reported that full‐body MMP7^KO^ mice do not perform terminal maturation (proteolysis) of pro‐defensins in PCs. Hence, MMP7^KO^ mice could be used as a mouse model without biologically active α‐defensins in PCs. Although the microbiota of these mice were shifted, these mice were healthy. However, they are less able to control infection with *Salmonella typhimurium*. Also, PC‐specific overexpression in mice of human α‐defensin 5 (*HD5*, considered human ortholog of the mouse α‐defensin genes) led to microbial shifts and protection against *S. typhimurium* challenge (Salzman *et al*, 2003). These data reflect the importance of α‐defensins in dealing with foreign bacterial invasions in the gastrointestinal tract (Wilson *et al*, 1999).

#### Bacterial signaling in PCs

**Figure 2:**
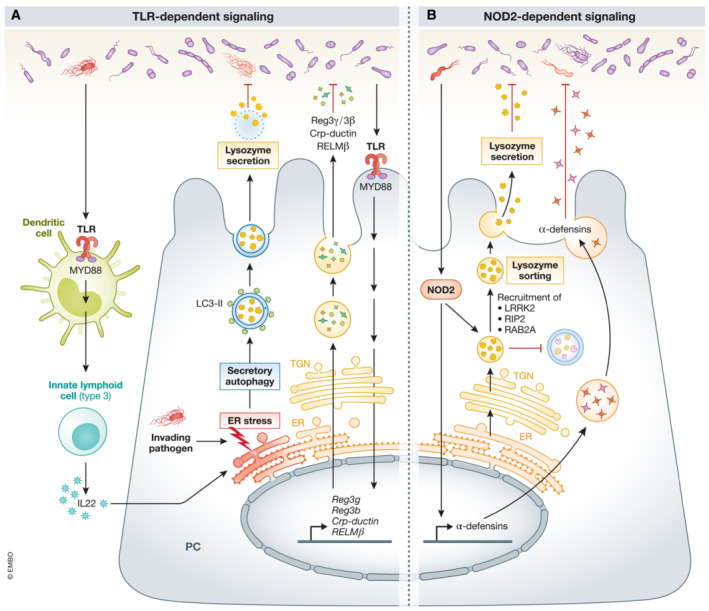
Bacterial stimulation of AMP expression and release in PCs (A) Intestinal bacteria can communicate directly with PCs *via* a PC‐autonomous TLR‐MYD88 pathway. This triggers the production and secretion of AMPs (measured in PCs by laser capture microdissection). There is also evidence for indirect extrinsic MyD88 bacterial signaling to activate the secretion of LYZ in PCs: *S. typhimurium* can stimulate dendritic cells *via* TLR‐MYD88 signaling, leading to IL22 production in innate lymphoid cells (type 3). IL22, together with invading *S. typhimurium* in the PC, induce ER stress in these cells, leading to secretory autophagy. The LYZ‐containing secretory autophagosomes have the typical features of an autophagosome, namely, a double membrane labeled with LC3. However, instead of fusing with lysosomes, these LYZ‐containing autophagosomes are released in the lumen of the small intestine. (B) Intestinal bacteria can stimulate NOD2 and increase α‐defensins production and secretion. Bacterial NOD2 activation can also lead to the recruitment of LRRK2, RIP2, and RAB2A onto the surface of DCSGs which coordinate lysozyme sorting.

The mechanism of bacterial sensing and signaling in PCs is incompletely understood. Yet, several pathways link bacterial signaling with increased expression and maturation of different AMPs (Fig 2). PCs might sense bacteria directly *via* myeloid differentiation primary response 88 (MYD88) and nucleotide‐binding oligomerization domain containing 2 (NOD2), which trigger the expression of a different subset of AMPs (Vaishnava *et al*, 2008; Dessein *et al*, 2009). But they might also be sensed indirectly by PCs, for example, *via* the MYD88 pathway in dendritic cells (Bel *et al*, 2017).

##### TLR/MYD88‐dependent bacterial signaling

In mice with genetic MYD88 deficiency, expression of several AMPs in PCs is reduced (*Reg3γ*, *Reg3*β, *CRP*‐*ductin*, and *RELM*) and bacterial translocation is increased (Vaishnava *et al*, 2008). Rescue of MYD88 expression only in PCs (by Defa2‐MyD88 transgenic expression in MYD88^KO^ mice) rescued this phenotype. These elegant experiments illustrated that (1) intestinal bacteria can communicate directly with PCs *via* cell‐autonomous MYD88 signaling and (2) the MYD88‐dependent antimicrobial response in PCs can prevent bacterial translocation. MyD88‐dependent pathways are essential for host defense against infections by *S. aureus*, *Toxoplasma gondii*, and *Listeria monocytogenes* (Scanga *et al*, 2002; Seki *et al*, 2002). There is also evidence for indirect extrinsic MyD88 bacterial signaling to activate the secretion of LYZ *via* secretory autophagy in PCs. Bel *et al* (2017) showed that *S. typhimurium* can invade PCs, damaging the Golgi apparatus and causing ER stress (Bel *et al*, 2017). This stress can activate secretory autophagy, only with the help of MyD88‐dependent DC signaling, followed by interleukin (IL) 22 production by innate lymphoid cells (type3). The LYZ‐containing secretory autophagosomes have the typical features of autophagosomes, a double membrane labeled with LC3. But, instead of fusing with lysosomes, these autophagosomes are released in the small intestinal lumen.

##### NOD2‐dependent bacterial signaling

α‐Defensin gene expression seems rather NOD2 dependent, as NOD2^KO^ mice have decreased expression of multiple α‐defensins (Kobayashi *et al*, 2005; Vaishnava *et al*, 2008; Tan *et al*, 2015). However, in contradiction with these previous reports, Menendez *et al* (2013) observed reduced α‐defensin expression in TLR2, TLR4, and MYD88^KO^ mice, but not in NOD2^KO^ mice (Menendez *et al*, 2013). LYZ secretion in PCs is also NOD2 dependent. It is synthesized in the ER of PCs and packed in dense core secretory granules (DCSG) at the trans‐Golgi network. Intestinal bacteria activate NOD2, leading to recruitment of leucine‐rich repeat kinase 2 (LRRK2), receptor interacting serine/threonine kinase 2 (RIP2), and RAB2A on the surface of these DCSGs, which coordinate LYZ sorting towards the lumen (Zhang *et al*, 2015; Wang *et al*, 2017a). Yet, other proteins may end up in lysosomes and become degraded. This LYZ‐sorting pathway is LYZ‐specific and does not apply to other AMPs.

#### Degranulation of AMP‐containing granules in PCs

The release of α‐defensins from the PC secretory granules can be activated by Gram‐negative or ‐positive bacteria, bacterial antigens (LPS, lipoteichoic acid, lipid A, and muramyl dipeptide), and carbamylcholine (Ayabe *et al*, 2000). This was shown by monitoring the antibacterial activity of *ex vivo* stimulated crypt cultures. Bacterial killing in the supernatant was not observed when crypts of MMP7^KO^ mice or mice devoid of PCs (CR2‐tox176) were stimulated with the mentioned ligands, emphasizing the antimicrobial role of α‐defensins in PCs. *In vivo* results showed that PCs are degranulated after stimulation with cholinergic agents, such as carbamylcholine, or by IL4, IL13, IL22, interferon (IFN)α, and tumor necrosis factor (TNF)α (Satoh *et al*, 1989; Ozcan *et al*, 1996; Rumio *et al*, 2012; Stockinger *et al*, 2014; Zwarycz *et al*, 2019). CpG‐oligodeoxynucleotides (TLR9 antagonists) and poly(I):poly(C) (TLR3 agonist) can also stimulate PCs, as they induce degranulation from 3 h postinjection. After oral treatment with LPS (a TLR4 agonist) and flagellin (a TLR5 agonist), late degranulation, mediated by TNFα, was observed in PCs (Rumio *et al*, 2012).

### Phagocytosis of bacteria and efferocytosis in PCs

Another remarkable function of PCs is that they can digest intestinal microorganisms *via* phagocytosis. Spiral‐formed bacteria and trophozoites of the flagellate *Hexamita muris* were identified in the digestive vacuoles of PCs from rats (Erlandsen & Chase, 1972). Both intact and partially digested spiral‐formed bacteria and trophozoites were observed, but only in PCs in the gut. The presence of *S. typhimurium* was also demonstrated in the PCs of infected mice (Bel *et al*, 2017).

Efferocytosis was recently identified as a new PC function (Shankman *et al*, 2021) that removes apoptotic cells by phagocytes. To illustrate that PCs can effectively engulf their neighboring apoptotic IECs, they made use of organoids derived from a transgenic mouse strain, where PC membranes were labelled green, and all membranes of all other cells red, in combination with apoptotic dyes. In enteroids irradiated to induce cell death, apoptotic IECs were engulfed by PCs. Also, PC‐specific ablation reduced efferocytosis in intestinal crypts. So, PCs can remove their neighboring apoptotic IECs and in this way reduce local inflammation and contribute to gut homeostasis (Shankman *et al*, 2021).

### Uptake of heavy metals

It has been established that PCs contain heavy metals (e.g., Se and Zn) (Danscher *et al*, 1985), along with heavy metal ion‐binding proteins, for example, metallothioneins and the Zn‐binding cysteine‐rich intestinal protein (Fernandes *et al*, 1997). PCs are believed to pick up heavy metals from the lumen and utilize them.

**Figure 3:**
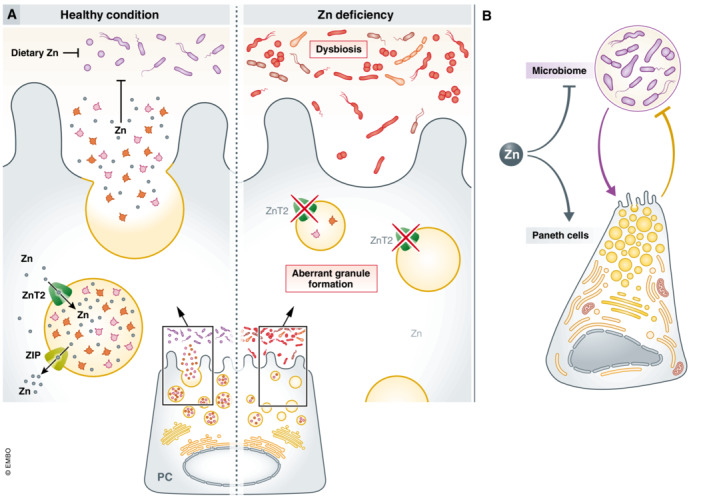
The direct and indirect effects (*via* PCs) of Zn on the microbiome (A) In healthy conditions, dietary Zn has a direct antimicrobial effect on the microbiome and inhibits growth of, for example, several *Staphylococcus* species. Moreover, ZnT2 is responsible for the accumulation of zinc in the secretory granules and for the regulation of AMP secretion in PCs. Upon degranulation, Zn is released in the lumen, leading to an indirect effect (*via* the PCs) of Zn on the microbiome. Moreover, *in vitro* assays suggest a role for Zn in stabilization of the antimicrobial HD5 and LYZ. In conditions of Zn deficiency, the direct and indirect antimicrobial effects of Zn on the microbiome are lost or reduced. Deletion of ZnT2 in mice leads to secretory granules devoid of Zn, impaired PCs, disturbed PC granule structure and reduced antimicrobial activity in the ileum, resulting in dysbiosis. (B) Zn can have an antimicrobial effect on the microbiome but is also indispensable for PC functions. PCs can further control the microbiome by the release of AMPs and Zn. The microbiome can in turn stimulate the PCs to produce AMPs.

Zn is essential for PC function, as deletion of some zinc importers/exporters causes PC defects. Fourteen Zn‐importing transporters (ZIPs encoded by *Slc39a* gene family members) and 10 Zn‐exporters (ZnTs, encoded by *Slc30a* family members) have been described, and several of them are expressed in PCs. An inducible loss‐of‐function of ZIP4 in the murine gut compromises PCs, causing abnormal expression of PC‐related genes (Geiser *et al*, 2012). Another Zn transporter, ZnT2, is responsible for exporting Zn towards the secretory granules. ZnT2 full KO mice (*Slc30a2* ^KO^) have impaired PCs devoid of Zn, disturbed PC granule structure and reduced antimicrobial activity in the ileum, leading to dysbiosis (Podany *et al*, 2016). These studies confirmed that Zn is not only directly antibacterial but also contributes to PC antimicrobial activities (Fig 3).

In PCs, Zn is stored in the secretory granules, but it is not clear why. One explanation is that Zn can stabilize HD5 and chicken egg LYZ, as shown *in vitro* (Chakraborti *et al*, 2010; Zhang *et al*, 2013). Both actions were confirmed *in vivo* in PCs (Podany *et al*, 2016; Zhong *et al*, 2020). It is also speculated that the storage of heavy metals contributes to direct antimicrobial toxicity, as Zn is released upon cholinergic PC stimulation (Giblin *et al*, 2006). A third potential function, only in mice, is that α‐defensin maturation is Zn‐dependent, as the final proteolytic α‐defensin maturation step is performed by MMP7, a Zn‐dependent metalloprotease (Wilson *et al*, 1999).

Moreover, Zn is important even in PC survival. In rats and mice, a single injection of the Zn chelator, dithizone, leads to disappearance of PCs and their reappearance after 12–24 h (Sawada *et al*, 1991). The PC‐specific ablation of PCs by dithizone indicates the strong Zn dependency of PCs.

### Preserving the intestinal barrier

Paneth cell‐derived AMPs were found to gather in the mucus to prevent invasion and microbial attachment, augmenting the antimicrobial function of the mucus (Meyer‐Hoffert *et al*, 2008). Moreover, PC‐deficient mice (Cryptdin2‐tox176) display increased bacterial translocation of commensal bacteria towards the mesenteric lymph nodes (Vaishnava *et al*, 2008), without adapting the luminal bacterial load in the small intestine (Vaishnava *et al*, 2008). This illustrates that PCs are essential in preventing bacterial translocation but have no effect on the overall bacterial load in the small intestine. A possible explanation is that PCs regulate the number of mucosa‐associated bacteria, which prevents close contact with the epithelium and subsequent bacterial translocation.

## PCs in pathology

Correct PC functioning is important for controlling the microbiota and preserving the crypt niche. This ensures the proper metabolic environment and ISC communication needed for tissue renewal (Lueschow & McElroy, 2020). Failure or disturbance in PC morphology and activity can reduce the secretion of AMPs and stemness factors and increase bacterial translocation. This determines the severity and progression of several gastrointestinal and other disorders in distant organs such as kidney or liver (Teltschik *et al*, 2012; Cray *et al*, 2021). Likewise, there is agreement that such diseases might be prevented or even cured by correcting PC abnormalities. Examples include inflammatory bowel disease, ileal CD, and necrotizing enterocolitis (NEC) (Barreto *et al*, 2022).

The PC dysfunction can be studied by quantifying the distribution and expression pattern of cytoplasmic AMPs, the morphology of the granules, and/or the number of PCs per crypt (Stappenbeck & McGovern, 2017). In this sense, genetic disorders, environmental factors, and diet are studied and linked as inducers of PC dysfunctions.

### PC numbers

It has been reported that PC numbers may decline by cell death and/or extrusion in the lumen, which aggravates intestinal and inflammatory diseases (Gassler, 2017). The reduction in PC number per crypt has been described in several pathological conditions, such as intestinal ischemia (Grootjans *et al*, 2011), pathogenic bacterial infections (White *et al*, 2017), NEC (White *et al*, 2017), and GVHD (Levine *et al*, 2013). However, in other intestinal diseases, such as ileal CD, the defect is in PC function and not in PC number (Deuring *et al*, 2014; Strigli *et al*, 2021). Moreover, changes in PC numbers are not always correlated with alterations in the expression of PC‐specific AMPs (Zwarycz *et al*, 2019; Kip *et al*, 2020). Newly formed PCs after an insult could be immature and/or dysfunctional and contain fewer or aberrant granules.

Paradoxically, in some cases, like alcohol‐fed animals or *Salmonella* infections, induction of cell death in the small intestine is coupled with increased PC numbers (Rodriguez *et al*, 2012). This could be explained by the fact that during intestinal inflammation, post‐mitotic PCs respond by acquiring stem cell‐like properties, thus contributing to the tissue regenerative response during inflammation (Schmitt *et al*, 2018). An increase in PC number per crypt has been observed after bacterial infection with *S. typhimurium* (Rodriguez *et al*, 2012). PC metaplasia has also been described in mouse models after infection (Singh *et al*, 2020). In humans, PC metaplasia has been described in NEC in premature infants (Puiman *et al*, 2011) and in CDs and ulcerative colitis patients (Tanaka *et al*, 2001).

Due to the location of PCs, the study of the number and function of these cells in humans is complex and requires tissue biopsies. The use of murine models is therefore fundamental to understanding the processes of the reduction in PC number and its association with different events.

### PC cell death

The ultimate fate of IECs, death, can occur by several mechanisms, either as part of normal physiology and homeostasis (cell renewal) or due to damage‐inducing events. PC death can be the result of physiological pathways defects, ER stress or autophagy, or by extrinsic cell death stimuli induced by factors such as cytokines.

#### Autophagy and unfolded protein response (UPR)

**Figure 4:**
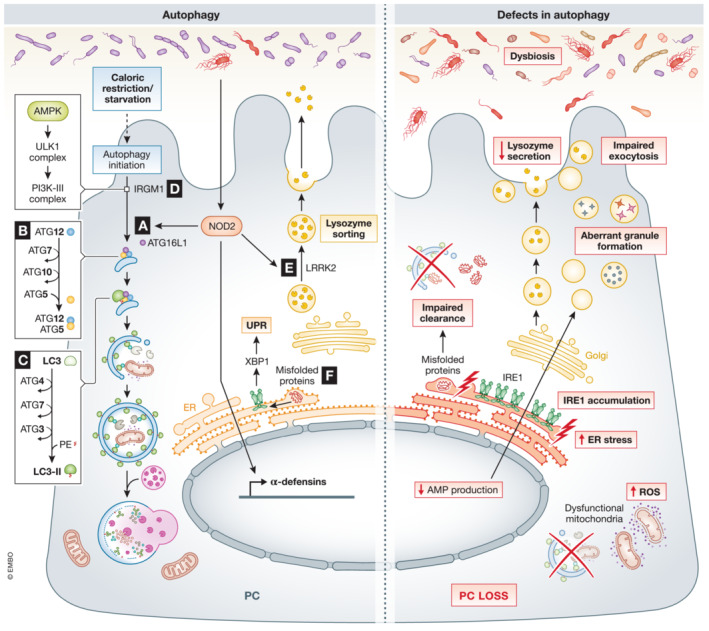
Role of autophagy and ER in PCs (A) Bacterial activation of NOD2 initiates direct autophagy by recruiting ATG16l1. Defects in *Nod2* and/or *Atg16l1* have been related to a decrease in AMP production, impaired exocytosis, accumulation of IRE1, and bacterial translocation. (B) In PCs, ATG5 binds ATG16L1 and ATG12, which is important in the early stages of autophagy, catalyzing the microtubule‐associated protein light chain 3 (LC3) lipidation. Specific deletion of *Atg5* in PCs has been associated with accumulation of ROS and increased ER stress due to impaired clearance of dysfunctional mitochondria. C) ATG4 and ATG7 form a complex with ATG3, responsible for the biogenesis of the autophagosome by determining the site of LC3 lipidation. Dysfunction in autophagy, aberrant granule formation, and defects in AMP production and secretion have been observed in *Atg4b* ^KO^ and *Atg7* ^*Δ*IEC^ mice. D) IRGM1 plays a direct role in organizing the autophagy process. IRGM1 can initiate the phosphorylation cascade that activates Unc‐51 like autophagy activating kinase 1 (ULK1) and Beclin1 (an autophagy regulator part of the PI3K‐III complex), which promotes autophagy. Defects in *Irgm1* have been linked to alterations in PC numbers and location, abnormal PC morphology and aberrant granules. E) LRRK2, together with RIP2 and RAB2A, coordinates lysozyme sorting after recruitment by bacterial‐activated NOD2. Dysfunctions in *Nod2* and/or *Lrrk2* result in compromised lysozyme secretion. F) Misfolded or unfolded proteins are recognized by IRE1, which by unconventional splicing generates XBP1, activating UPR response. Specific deletion of *Xbp1* in PCs leads to PC dysfunction, a condensed ER and abnormal secretory granules. Black arrows – pathways that are active in PCs, red arrows – “up‐ or downregulation” of factors that negatively affect PCs function.

In PCs, autophagy is important to manage the generation and exocytosis of the secretory granules, clearance of misfolded proteins, maintenance of mitochondrial homeostasis, alleviation of ER stress, bacterial autophagy, and survival cytokine‐mediated immunopathology after infections (Burger *et al*, 2018; Wang *et al*, 2018). Defects in autophagy or autophagy related‐genes have been linked to abnormalities in PC morphology and function (Table 3; Fig 4) (Wang *et al*, 2018).

**Table 3:** Mouse studies linking defects in autophagy and/or unfolded protein response (UPR)‐related genes with abnormalities in PCs' morphology and function.

	Knocked‐out gene	Cell type	Mechanism	Outcome	References
UPR‐related genes	X‐box‐binding protein 1 (*Xbp1*)	Intestinal epithelial cells	Condensed ER ER stress Abnormal secretory granules Decreased expression of alpha‐defensins Paneth cell apoptosis	Susceptibility to DSS‐induced colitis and bacterial infections	Kaser *et al* (2008)
		Paneth cells	Autophagy activation Abnormal secretory granules Increased cell death in crypts Unresolved ER stress UPR activation	Transmural ileitis Development of spontaneous intestinal inflammation	Adolph *et al* (2013)
		Intestinal epithelial cells	Increased total and phosphorylated IRE1α Increased NFκΒ activity Autophagosome formation Aberrant Paneth cell granules	Ileitis dependent on microbiota	Adolph *et al* (2013)
Autophagy‐related genes	*Atg16l1*	Intestinal epithelial cells	Crypts with increased XBP1 splicing Reduction in Paneth cell size and number of granules Loss of homeostatic autophagy	Susceptibility to DSS‐induced inflammation	Adolph *et al* (2013)
		Intestinal epithelial cells	Accumulation of IRE1α in Paneth cells	IRE1α‐dependent spontaneous ileitis	Tschurtschenthaler *et al* (2017)
		Intestinal epithelial cells	Paneth cell depletion	Exacerbated intestinal injury in models of graft‐versus‐host disease	Ishimoto *et al* (2017)
	*Atg16l1* ^HM^ (hypomorphic (HM) for expression of the ATG16L1 protein)	Constitutive	Aberrant Paneth cell granules (size, morphology, and number) Irregularities in the granule exocytosis pathway Morphological abnormalities in Paneth cells Diffuse lysozyme staining		Cadwell *et al* (2008)
	*Atg16l1* ^*T300A*^ (Thr^300^ ➔ Ala^300^)	Constitutive	Abnormal Paneth cell lysozyme distribution and decreased antibacterial autophagy after infection		Bel *et al* (2017)
	*Atg16l1/Xbp1*	Intestinal epithelial cells	Lack of UPR‐induced autophagy Increased total and phosphorylated IRE1α Increased NFκB activity Aberrant Paneth cell granules	Development of severe spontaneous ileitis Transmural inflammation	Adolph *et al* (2013)
		Intestinal epithelial cells	Impaired clearance of IrE1α aggregates ER stress Impaired Paneth cell antimicrobial function	Aggravated DSS‐induced colitis	Tschurtschenthaler *et al* (2017)
	*Atg5*	Paneth cells	Abnormalities in number and size of Paneth cell granules Reduced expression of AMPs	Paneth cell loss upon *T. gondii* infection Increased permeability due to impaired intestinal barrier function	Burger *et al* (2018)
		Intestinal epithelial cells	Reduced expression of AMPs Impaired autophagy in Paneth cells	Susceptibility to *T. gondii*‐mediated intestinal damage Paneth cell loss upon *T. gondii* infection	Burger *et al* (2018)
		Intestinal epithelial cells	Morphological abnormalities in Paneth cells Irregularities in the granule exocytosis pathway Aberrant Paneth cell granules (size, morphology, and number)		Cadwell *et al* (2008), Cadwell *et al* (2009)
	*Atg7*	Intestinal epithelial cells	Deficient Paneth cell‐granule formation Alterations in lysozyme storage and secretion		Wittkopf *et al* (2012)
		Intestinal epithelial cells	Morphological abnormalities in Paneth cells Aberrant Paneth cell granules (size, morphology, and number) Diffuse lysozyme staining		Cadwell *et al* (2009)
	*Atg7/Xbp1*	Intestinal epithelial cells	Absent UPR‐induced autophagy Aberrant Paneth cell granules	Spontaneous ileitis Transmural inflammation Age‐dependent enteritis	Adolph *et al* (2013)
	*Atg4b*	Constitutive KO	Abnormalities in size, morphology, and number of Paneth cell granules Autophagy impairment	Increased susceptibility to DSS‐induced colitis	Cabrera *et al* (2013)
	Leucine‐rich kinase 2 (*Lrrk*2)	Constitutive KO	Defects in lysozyme sorting	Increased susceptibility to intestinal *Listeria monocytogenes* infections	Zhang *et al* (2015)
	Immunity‐related GTPase M (*Irgm1*)	Constitutive KO	Alterations in Paneth cell numbers and location	Susceptibility to dextran sodium sulfate (DSS)‐induced intestinal injury	Rogala *et al* (2018)
		Constitutive KO	Decreased transcript levels of specific AMPs (*Lyz* and *Defa20*) Alterations in Paneth cell numbers and location Aberrant Paneth cell‐granules (size and morphology) Abnormal Paneth cell morphology Impaired mitophagy and autophagy	Spontaneous intestinal inflammation Ileal injury	Liu *et al* (2013)
	Transcription factor EB (*Tfeb*)	Intestinal epithelial cells	Abnormal morphology of Paneth cell granules	Magnified colitis response upon DSS	Murano *et al* (2017)

An important gene involved in autophagy is the autophagy‐related 16 like 1 gene (*Atg16l1* in mice, *ATG16L1* in humans). The importance of *Atg16l1* is mainly in its participation in the formation of autophagosomes, but its function has been linked with other major cell activities. A study on PC‐rich organoids isolated from WT and *Atg16l1*‐deficient mice showed significant differences in their proteomic signature. In KO cells, 16 important functional processes were altered, the most remarkable being inhibition of the exocytosis pathway (Jones *et al*, 2019). According to one study, mice with *Atg16l1* and *Atg5*‐deficient‐PCs presented irregularities in the granule exocytosis pathway, which is observed in CD patients with the homozygous ATG16L1 risk allele (*Atg16l1* ^*T300A*^) (Cadwell *et al*, 2008). KI mice with *Atg16l1* ^T300A^ have a similar phenotype, with abnormalities in PC granules, function, and PC numbers (Lassen *et al*, 2014). In mice, villin or PC‐specific deletion of *Atg16l1*, *Atg5* or *Atg7*, or constitutive deletion of *Atg4b* or immunity‐related GTPase family M member (*Irgm1*) result in impaired exocytosis, accumulation of dysfunctional mitochondria, accumulation of inositol‐requiring enzyme 1 (IRE1) and decrease in AMP production (Fig 4A–D). Likewise, mutations of *Lrrk2* led to degradation of LYZ *via* autophagy (Fig 4E). PC‐specific deletion of *Lrrk2* has also been linked with the abnormal PC‐phenotype seen in Japanese CD patients (Liu *et al*, 2017b).

Paneth cells, due to their intense secretions, are very sensitive to ER stress, making autophagy and the UPR important in preventing PC death. The heavy load on the ER network in PCs makes them more prone to ER stress than other cells. Disruption of PC secretions or defects in UPR‐related genes might lead to dysbiosis and inflammatory diseases (Bel *et al*, 2017; Wang *et al*, 2018). Deletion of the transcription factor X‐box‐binding protein 1 (*Xbp1*) specifically in PCs leads to unresolved ER stress, autophagy activation, and PC apoptosis (Table 3; Fig 4F) (Kaser *et al*, 2008; Adolph *et al*, 2013). Induction of PC‐specific ER stress has also been observed in SAMP1/YitFc mice, which are a model of CD (Shimizu *et al*, 2020). The disruption of ER homeostasis in those mice leads to α‐defensin misfolding associated with absence of disulfide bridges, and dysbiosis linked to progression of ileitis. The absence of disulfide bridges in HD5 (leading to reduced rather than oxidized HD5) was observed in PCs of CD patients (Tanabe *et al*, 2007). Also in CD patients, an increased abundance of enteroinvasive *Escherichia coli* and other signs of dysbiosis were associated with ER stress in PCs (Deuring *et al*, 2014).

#### Cell death by ligands: IFNs

Interferons have been postulated as critical modulators of PC function and survival (Araujo *et al*, 2021). A recurrent observation is that IFNs induce PC cell death. IFNs I, II, and III have been linked to PC death and/or degranulation (Günther *et al*, 2019; Araujo *et al*, 2021). Why PCs are so IFN‐sensitive and the mechanisms of their loss are unknown. However, we will explain that the effects of IFN on PCs are not necessarily direct and that indirect effects are contemplated.

**Figure 5:**
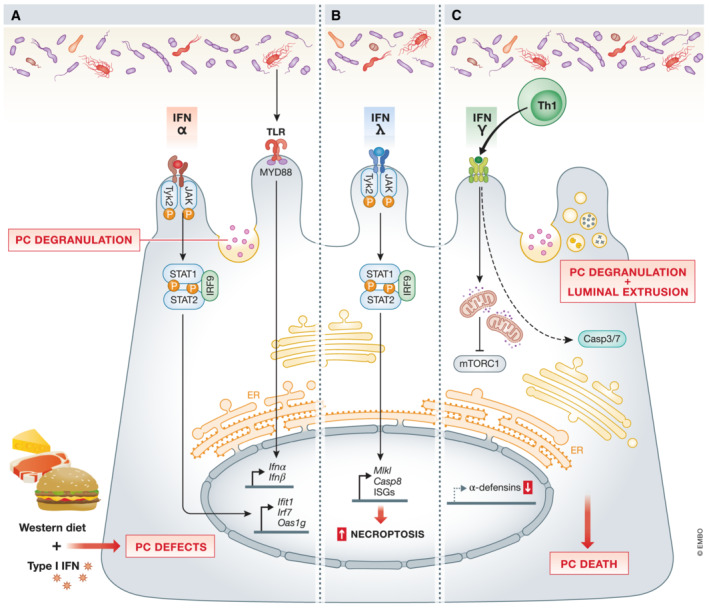
Role of the three types of IFNs in PCs (A) IFNα regulates PC‐numbers *via* JAK/STAT signaling. The release of type I IFN can also be triggered by bacterial‐TLR activation. The combination of Western diet and type I IFN, produced by myeloid cells, negatively affects PCs (see Figure 6 for more details). (B) Detrimental effects have been ascribed to type III IFNs, as IFNλ promotes PC necroptosis in an MLKL/CASP8‐dependent manner. (C) Schematic representation of the mechanisms related to PC‐loss associated with type II IFN (IFNγ). IFNγ mediates PC loss by an mTORC1‐dependent mechanism after disrupting mitochondrial integrity and function. Moreover, after injury, activated Th‐produced IFNγ induces PC depletion *via* a caspase3&7‐dependent cell death. Black arrows – pathways that are active in PCs, red arrows – “up‐ or downregulation” of factors that negatively affect PCs function.

Type I‐IFNs (mainly IFNα and IFNβ) are involved in antimicrobial host defense due to their antiviral and antiproliferative activities. They are considered as essential in the first line of defense against viruses (Liu *et al*, 2012) and bacteria and are triggered by stimulation of TLRs (Decker *et al*, 2005) and other pathogen sensors. In PCs, TLR9 signaling might be responsible for the bacterially triggered type‐I IFN production (Fig 5A). Through the IFN I receptor (a heterodimer consisting of IFNAR1 and IFNAR2), type I IFNs can activate several Janus kinases (JAKs), several signal transducers and activators of transcription (STAT), and MAPK signaling pathways (González‐Navajas *et al*, 2012). Although PCs constitutively express *Ifna1* (encoding IFNα1) under homeostatic conditions, little is known about its biological effect. Feeding mice with the IFN‐inducing compound, R11567DA, induced IFNα in ileum, colon, and blood, but in situ hybridization (ISH) experiments showed that IFN‐dependent genes were only induced in crypts, likely in PCs (*Ifit1*, *Irf7*, and *Oas1g* genes). Perhaps PCs are unique in expressing *Ifnar1*, or the signal transduction to IFNs in PCs is much more pronounced because of the lack of inhibitors (Munakata *et al*, 2008). In another study, expansion in PC numbers in mice lacking the IFNAR1 in the gut (*Ifnar1* ^*Δ*IEC^) was observed, meaning that IFNα can regulate PC numbers (Tschurtschenthaler *et al*, 2014). Finally, IFNα injection in Wistar rats was shown to cause PC degranulation and α‐defensin release (Ozcan *et al*, 1996).

The type III IFN group, consisting of four IFNs generally termed IFNλ, is mainly sensed by epithelial cells. Günther *et al* (2019) reported that in the gut epithelium, IFNλ causes the expression of mixed lineage kinase domain like pseudokinase (*Mlkl*) and *Stat1*, which leads to increased necroptosis in PCs, controlled by caspase8 (CASP8) (Fig 5B) (Günther *et al*, 2019). This increased PC death has been seen in CD patients, in whom also higher levels of IFNλ were observed in serum and inflamed ileal tissue (Stolzer *et al*, 2021).

Recent findings show that of all IFNs, type II IFN (IFNγ) is the most harmful: by promoting PC loss, it is involved in intestinal inflammation (Fig 5C) (Raetz *et al*, 2013; Farin *et al*, 2014; Eriguchi *et al*, 2018). *In vitro* and *in vivo* studies have shown that IFNγ hampers the expression of α‐defensins, increases degranulation, and leads to PC death (Bevins & Salzman, 2011; Raetz *et al*, 2013; Farin *et al*, 2014; Burger *et al*, 2018; Eriguchi *et al*, 2018; Araujo *et al*, 2021). In serum samples and ileal biopsies of IBD mouse models, increased IFNγ concentrations are concomitant with a higher rate of PC loss (Wang *et al*, 2018). Moreover, ileal samples from *T. gondii*‐infected mice had significantly fewer PCs. This occurred entirely though events in the submucosa, namely, dendritic cell activation (Th1 activation) *via* IL12. IFNγ production by these Th cells ultimately led to PC death (Pêgo *et al*, 2019), a phenotype that was reverted in mice with an epithelial or PC‐restricted IFNγRII KO (Araujo *et al*, 2021). Though it seems clear that IFNγ leads to PC death, the mechanisms remain uncertain, particularly whether this loss in PCs is a direct or indirect effect of IFNγ. Experiments on a GVHD mouse model have shown that alterations in the crypt cell niche, and consequently in PCs, were due to effects of IFNγ on ISCs (Takashima *et al*, 2019). Yokoi *et al* (2021) showed that both PCs and ISCs express IFNγRI, and that IFNγ caused cell death directly in both cells types. The same was proposed by Eriguchi *et al* (2018), who observed that IFNγ selectively induced PC death, which might impact the whole intestinal crypt (Eriguchi *et al*, 2018). While some authors proposed that IFNγ is sufficient to mediate PC loss (Farin *et al*, 2014; Eriguchi *et al*, 2018), others reported that both the microbiota and the basal microbiota‐induced IFNγ are needed to cause intestinal pathologies and sustained PC death in *Atg5*‐deficient mice (Burger *et al*, 2018). Recent findings have supported the idea that PCs can undergo different mechanisms of death when exposed to IFNγ. The study carried out by Farin *et al* (2014) confirmed that IFNγ‐induced PC death was caspase3 and 7‐dependent (Farin *et al*, 2014; Eriguchi *et al*, 2018). However, others stated that PC death was induced by the inhibition of mTORC1 by IFNγ (Araujo *et al*, 2021). Further investigations have confirmed that the decreased levels in mTORC1 resulted from impaired mitochondrial function as an indirect response to IFNγ (Araujo *et al*, 2021). Although autophagy can alleviate the effects of mitochondrial accumulation, it seems insufficient to rescue the loss of PCs, probably due to excessive autophagy or the prolonged inhibition of mTORC1. Thus, IFNγ‐stimulated effects might be exacerbated when autophagy defects exist (Ishimoto *et al*, 2017).

We speculate that IFNs might be so cytotoxic to PCs because PCs are direct neighbors of ISCs. As PCs sustain the continuous renewal of the intestinal layer, one of their main functions could be the protection of ISCs. They may be programmed to (1) be extremely attractive to viral infection and titrate viruses away from the ISCs, and (2) die and thereby release antimicrobial peptides/proteins to control potential bacterial superinfections of the crypts. In this view, we argue that the loss of PCs associated with potential local dysbiosis may be less damaging than the loss of ISCs.

#### Cell death by ligands: TNF

TNF also plays an important role in autoimmune and inflammatory disorders (Puimège *et al*, 2014; Van Looveren & Libert, 2018). *In vivo* studies have shown that TNF injection can lead to systemic inflammation characterized by the secretion of proinflammatory cytokines and can induce cell death. Interestingly, in the small intestine, TNF induces apoptosis by binding exclusively to TNF receptor 1 (TNFR1) (Van Hauwermeiren *et al*, 2015; Ballegeer *et al*, 2018; Van Looveren *et al*, 2020). TNF can bind two distinct receptors, namely TNFR1, which mediates most TNF effects, and TNFR2. After TNF is recognized by TNFR1, it can activate proinflammatory and pro‐survival NF‐κB pathways or induce cell death by Fas‐associated death domain (FADD)‐CASP8‐dependent apoptosis or RIPK3‐MLKL‐mediated necroptosis (Pasparakis & Vandenabeele, 2015). The study conducted by Van Hauwermeiren *et al* (2015) showed that TNF injection in mice induced PC dysfunction characterized by an increase of ER stress, with disturbed and dilated morphology. Moreover, PCs had fewer granules, lost cellular integrity, and became dysfunctional, which may lead to an increase in bacterial translocation induced by TNF (Van Hauwermeiren *et al*, 2015).

Over recent years, different KO mouse models of genes involved in the TNF‐induced pathways have been generated to study their role and the PC status. Specific ablation of NF‐κB essential modulator (NEMO) in intestinal cells caused PC apoptosis and impairments in AMP expression (Vlantis *et al*, 2016). Interestingly, the effects of NEMO^ΔIEC^‐mediated PC loss were independent of microbiota, as it was also seen in GF mice, but the lack of bacterial translocation ameliorates the phenotype. In IEC‐specific KO of NF‐κB subunit RelA, the absence of *Ripk1* protected mice from PC death (Vlantis *et al*, 2016). Also, intestinal and myeloid deletion of A20 (TNFAIP3, encoded by *Tnfaip3* in mice, *Tnfaip3* ^ΔIEC/Δmyel^), an inhibitor of NF‐κB and apoptosis, induces ileitis and severe colitis characterized by IEC apoptosis, goblet cell, and PC loss. The specific deletion of *Tnfaip3* only in epithelial cells (*Tnfaip3* ^ΔIEC^) does not have a pathological phenotype but sensitize these mice to DSS‐induced colitis and TNF‐induced apoptosis: 24 h after injection of a sublethal TNF dose, LYZ‐containing PCs and expression of PC‐specific AMPs were reduced (Vereecke *et al*, 2014). Those experiments were confirmed in *Tnfaip3*‐deficient organoids which were more affected than WT organoids by TNF combined with IFNγ (Vereecke *et al*, 2014). In contrast, complete ablation of *Nf‐κb* in intestinal cells causes less severe PC loss, as observed in NEMO^ΔIEC^ mice, suggesting that pathways besides NF‐κB are involved in prevention of PC death (Vlantis *et al*, 2016).

In CASP8^ΔIEC^ mice and FADD^ΔIEC^ mice, cell death in the intestinal crypts increased spontaneously, especially in PCs (Welz *et al*, 2011; Günther *et al*, 2012). FADD is one of the main regulators of CASP8‐mediated inflammatory responses. RIPK1 and RIPK3 have found to be crucial in PC death, as deletion of these regulators lead to protection in FADD^ΔIEC^ mice (Welz *et al*, 2011; Schwarzer *et al*, 2020). Particularly in CASP8^ΔIEC^ mice but also in FADD^ΔIEC^ mice, inhibition of RIPK1 kinase was as protective as the combined deficiency of TNFR1 and Z‐DNA‐binding protein 1 (ZBP1), indicating that the role of RIPK1 is downstream of TNFR1 and ZBP1 activation (Schwarzer *et al*, 2020). The study by Schwarzer *et al* (2020) identified a key role of ZBP1, which appears to have similar functions as TNFR1 in the ileum. Therefore, only the double mutant can partially rescue PC loss and ileitis in FADD^ΔIEC^ mice. Günther *et al* (2012) identified a role for CASP8 in protecting IECs from TNF‐induced RIPK3‐mediated necroptosis. Increased PC death was observed in the ileum of CD patients, and *ex vivo* treatment with TNF on human control biopsies reduced *Lyz* in PCs, which can be rescued by an inhibitor of necroptosis. Moreover, in PCs, RIPK3 is present specifically in terminal ileum samples of human patients, but not in other intestinal cell types. These results indicate that high levels of exogenous TNF in the lamina propria, as in CD patients, can induce PC necroptosis and deficient expression of antimicrobial peptides, which may add to disease progression (Günther *et al*, 2012).

So, deficiencies in genes related to apoptosis sensitize PCs to TNF. Loss of X‐linked inhibitor of apoptosis protein (XIAP) was recently reported to sensitize to TNF‐ and microbiota‐dependent intestinal inflammation (Strigli *et al*, 2021; Wahida *et al*, 2021). Deficiency in XIAP has been linked to NF‐κB impairment, alteration in the direct binding and inhibition of caspases, and altered recognition of bacteria, the last due to the critical role of XIAP in the NOD1 and NOD2 complex (Strigli *et al*, 2021). Exacerbation and development of intestinal inflammation in this mouse model were specifically due to the negative effects in PCs. Impaired expression of α‐defensins‐PC, aberrant granules, and reduced PCs numbers were observed in the ileum of *Xiap* ^KO^ mice and *Xiap* ^ΔRING^ mice (lacking the C‐terminal RING domain of XIAP). Further crosses with TNFR1^KO^ and RIPK3^KO^ mice prevented PC loss in *Xiap* ^KO^ mice, demonstrating that ablation of XIAP promotes TNF‐ and RIPK1/3‐dependent PC death (Strigli *et al*, 2021). Variants in *Ripk1* and *Casp8* are observed in patients with severe immune deficiencies and IBD (Wahida *et al*, 2021).

Mice with a deletion in the TNF AU‐rich elements (TNF^ΔARE^) suffer from CD‐like ileal inflammation due to loss of translational control of TNF linked to PC dysfunction. This dysfunction was characterized by decreased expression of *Lyz1* and *Defa5*, abnormal PC granularity and reduction in PC numbers. Confirming the phenotype, TEM analysis showed that remaining PCs have aberrant secretory granules, enlarged rough ER, and degenerative mitochondria, the latter being key in the CD‐associated loss of stemness (Khaloian *et al*, 2020).

These findings indicate that deletion of specific genes involved in autophagy, MAPK and NF‐κB signaling, and apoptosis, might lead to defective PCs, increase susceptibility to infection and/or IFN‐/TNF insults, or even to cell death, in some cases specifically in PCs.

### PCs in disease

Paneth cell (PC) dysfunction or reduced numbers per crypt have been observed in several inflammatory, infectious, or rejection diseases. CD‐associated *E. coli* is able to penetrate deep in the intestinal crypts, but the link between reduced numbers of PCs, PC function, or increased resistance of the bacteria to α‐defensins is not yet clear. Enterotoxigenic *E. coli* (ETEC) infection led to ileal inflammation and fewer PC markers (Ren *et al*, 2014). Mortality of ETEC‐infected mice was about 30% after 24 h, and PC markers (measured by qPCR) were reduced in the ileum (Liu *et al*, 2017a). In a mouse model of *S. typhimurium* infection, PC death was associated with bacterial translocation to the spleen. The PC death was inhibited by treatment with *Lactiplantibacillus plantarum*. This effect of *Lpb. plantarum* might have been due to inhibition of pathogen colonization by competitive exclusion or mediated by TLR4/NF‐κB activation (Ren *et al*, 2022).

Recent studies have shown that abnormal PC morphology and/or decreases in α‐defensins are present in 50% of pediatric CD patients (Perminow *et al*, 2010; Liu *et al*, 2018). As dysfunction in autophagy‐related genes trigger PC dysfunctions, it is not surprising that autophagy defects have been correlated with PC aberrations in CD patients (Table 3). Investigators recently reported that abnormalities in PCs increase the risk of ileitis and CD, and consider PCs as central players in the ileal chronic inflammation in CD patients (Wehkamp & Stange, 2020; Cray *et al*, 2021). Some genes (e.g., *Nod2*, *Atg16l1*, *Atg5*, *Xbp1*, and *Lrrk2*) described in Table 3, are identified as risk factors for CD. Moreover, ileal CD patients carrying mutations in some of these genes (e.g., *Atg16l1*, *Nod2*, and *Lrrk2*) have reduced expression of α‐defensins or PC abnormalities. These results support the idea that PC functional problems are the initiators of ileal CD in patients. Moreover, in some cases those effects might be aggravated by non‐specific consequences of an environmental trigger (e.g., diet, antibiotics, and tobacco) (Wehkamp *et al*, 2007; Stappenbeck & McGovern, 2017). Yet, it is still debated if, and if so, how PC dysfunction can affect inflammation in the colon. A possible explanation is that in healthy conditions, functional α‐defensins from the ileum are found in the colon in an active form, where they can exert an effect (Mastroianni & Ouellette, 2009). Moreover, metaplastic PCs in the colon in IBD are considered as a repair mechanism and a useful marker of the disease (Tanaka *et al*, 2001).

Paneth cells have also been identified as sensors of viral infection. A study in male rhesus macaques infected with simian immunodeficiency virus (SIV) showed a correlation between epithelial damage and induction of IL‐1b expression by PCs preceding the antiviral interferon host response (Hirao *et al*, 2014). That study reported changes in the microbial composition of the macaques (after the infection) that may promote opportunistic enteric bacterial infections (Zaragoza *et al*, 2011). Moreover, a recent study with transmissible viral gastroenteritis highlighted the negative impact of PC loss, caused by the infection, and the inhibition of Notch factors secretion on ISC self‐renewal and differentiation (Wu *et al*, 2020).

In human obese patients, a shift in the microbiome, activation of the UPR in the gut, and reduced HD5 and LYZ in PCs were observed (Hodin *et al*, 2011b). Moreover, PC numbers were reduced in a human model for ischemia/reperfusion (Grootjans *et al*, 2011). In mice, clear relations between pathology, reduced PC number, and dysbiosis have been found in a model of GVHD. The rejection of tissue in the mouse model led to changes in microbiota composition. In GVHD, a convincing role of PCs in the pathology has been demonstrated, because PC numbers were greatly reduced in GVHD mice, declining from five to six PCs per crypt to just one PC per crypt. Then, *E. coli* colonization in the gut increased, followed by tissue invasion and death. The data demonstrated, indirectly, the critical role of PCs in controlling the pathological impact of graft‐initiated inflammation and immune reactions (Ara & Hashimoto, 2021). Cytokines such as IL22 or proteins like R‐spondin‐1 showed success in preventing dysbiosis and improving PC status in *in vivo* models of GVHD (Takashima *et al*, 2011; Hanash *et al*, 2013; Hayase *et al*, 2017).

### Other cytokines

Takahashi *et al* (2008) were among the first to report that PCs can produce IL17 under certain inflammatory conditions, like TNF challenge. IL17 production was shown to be PC‐specific in the gut (Takahashi *et al*, 2008). This increase in TNFα‐induced IL17 drives further the intestinal inflammation and is responsible for the damage observed in the small intestine (Takahashi *et al*, 2008). Others showed in mice that alcohol led to ER stress‐mediated IL17 production in PCs, which increased apoptosis and permeability, leading to bacterial translocation. Normality was restored by antibody blockage of IL17A (Gyongyosi *et al*, 2019). Moreover, the effects of PC‐induced IL17A have been linked to multiorgan dysfunction (Takahashi *et al*, 2008; Park *et al*, 2011, 2012). Remarkably, PCs, and not Th17 cells, have been described as the main source of IL17A, and this was also shown by the decrease of IL17A after PC ablation (Park *et al*, 2011; Gyongyosi *et al*, 2019). On the contrary, the use of IL17A inhibitors in CD and ulcerative colitis patients increased the adverse effects (Hueber *et al*, 2012). Since IL17 receptors are ubiquitously expressed, IL17 produced by PCs could be considered as a locally produced product that has systemic amplifying activities and might be considered as a therapeutic target in systemic diseases.

The roles of other cytokines, such as IL22, in PC maturation and function were recently studied (Mühl & Bachmann, 2019; Gaudino *et al*, 2021; Chiang *et al*, 2022). Early observations showed that loss of IL22 in the PCs of mice significantly compromises AMP production. A recent study published in Nature highlighted the crucial STAT3‐dependent role of IL22 in crypt regeneration and PC maturation, and pointed to the activation in PCs of IL22–STAT3 signaling response after adherent‐invasive *E. coli* infection (Chiang *et al*, 2022). These findings have been confirmed in organoids: production and secretion of LYZ, as well as other PCs markers, increased after IL22 supplementation (Zwarycz *et al*, 2019; Gaudino *et al*, 2021). In addition, IL22 alleviates high‐fat diet effects by improving the status of PCs and increasing the production of AMP (Gaudino *et al*, 2021). Moreover, IL22 reverted the decrease of Reg3γ in PCs in a mouse model of GVHD (Zhao *et al*, 2018). However, studies on organoids have shown that persistent IL22 signaling can negatively impact PCs by increasing ERS, leading to a decrease in the numbers of ISCs and PCs (Zhao *et al*, 2018). Downstream of IL22–STAT3 activation is an IL18–IFNγ cascade, which might contribute to host defense against infections such as by adherent‐invasive *E. coli*. Cooperation between IL22 and IL18 in a coordinated inflammatory response has been previously described in other cell types (Mühl & Bachmann, 2019). While IL22 seems to activate the cascade in response to an infection, IL18 is indispensable for the PC response and homeostasis. IL18^KO^ mice displayed fewer LYZ‐containing PCs and appeared to be more sensitive to bacterial infections. Moreover, organoids increased their AMP production when stimulated with IL18 (Chiang *et al*, 2022). However, there is no agreement about the role and the expression of *Il22r* and *Il18r* in PCs. Gaudino *et al* (2021) showed that PC maturation and functions depend on cell‐intrinsic IL22Ra1 signaling, and this PC‐specific IL22Ra1 signaling provide immunity against *S. typhimurium* (Gaudino *et al*, 2021). However, Chiang *et al* (2022) showed that the expression of *Il22r* is specific to Lgr5+ ISCs, and that the expression of *Il18r* is clearly higher in PCs (Chiang *et al*, 2022). Due to the participation of IL18 and IL22 receptors in PC antimicrobial response and homeostasis, it is important to unravel their expression profiles and mechanisms of activation.

### PCs and aging

Epithelial homeostasis also depends on the balance of the ISC response between self‐renewal and differentiation. The correct management of this continuous cell renewal is crucial for the intestinal epithelium's functions in absorption of nutrients and hormone secretion, while discriminating between commensal and pathogenic microbes.

In the small intestine, a decline in mucosal renewal during aging might lead to a defective immune system and an increased susceptibility to infections (Pentinmikko & Katajisto, 2020). Several authors have reported that the regenerative potential of human and mouse intestinal epithelium decreases with age due to defects in ISCs and in their niche (Nalapareddy *et al*, 2017). As stem‐cell‐niche supporters, PCs play a key role in tissue regeneration, regulating the number and function of ISCs by secreting niche‐signaling factors, and their metabolism by producing lactate (Sato *et al*, 2011a; Rodríguez‐Colman *et al*, 2017). Several studies have reported that during aging, the size and cellular composition of the crypts changes (Pentinmikko & Katajisto, 2020; Nalapareddy *et al*, 2022). Notably, the number of PCs increases in aged mice and humans, but conflicting results were reported on Lgr5+ cell numbers (Moorefield *et al*, 2017; Nalapareddy *et al*, 2017; Mihaylova *et al*, 2018; Pentinmikko & Katajisto, 2020). *In vitro* studies on aged ISCs cells and crypts from aged humans and mice have shown impaired regeneration. The impairment and reduction of the regeneration activity of ISCs in aging might be due to an increased expression in *Atoh1*, and a decrease of both *Olfm4* and *Notch1* compared with young ISCs (Nalapareddy *et al*, 2022).

Experiments on organoid cultures were used to establish the role of young and old PCs in the maintenance of the niche homeostasis (Nalapareddy *et al*, 2017; Pentinmikko *et al*, 2019). Generation of organoids from isolated ISCs and PCs have shown that the signals specifically from aged PCs compromised the niche function, probably due to reduced canonical Wnt signaling, that might be related with PC dysfunction (Pentinmikko *et al*, 2019). However, little is known about the overall function and morphology of old PCs. Differences in gene expression has been reported between old and young PCs, such as the reduced expression of *Wnt3a* (Nalapareddy *et al*, 2017). Indeed, *in vitro* supplementation with Wnt3a to aged human and murine organoids improved regeneration of old epithelia (Nalapareddy *et al*, 2017). This functional decline in stemness‐maintaining Wnt signaling may be due to the production of Notum, a negative extracellular Wnt regulator produced only in aged PCs (Yilmaz *et al*, 2012). One of the main reasons for this increase in Notum activity might be the activation of mTORC1. Mechanistically, high activity of mTORC1 in aged PCs inhibits the activity of peroxisome proliferator‐activated receptor α (PPARα), which ultimately leads to an increase in the expression of *Notum* (Pentinmikko *et al*, 2019). The importance of Notum was confirmed *in vitro* and *in vivo* by using ABC99, an inhibitor of Notum, which restored the Wnt‐mediated PC and ISC functions, leading to increased regeneration activity in old crypts comparable to those from young mice (Pentinmikko *et al*, 2019). However, little is known about the functional effect of the bias towards the production of more PCs on the intestinal crypts and the influence on tissue regeneration (Nalapareddy *et al*, 2022). Why do aged intestines need more PCs? And what is their function in aged subjects?

Another explanation for alteration in PC numbers during aging might also be the lowered Zn availability. Several studies have shown that aging in people is associated with a decrease in blood Zn levels (Meunier *et al*, 2005). This may be partly due to changes in dietary choices and feeding behavior but could also be a reflection of reduced uptake of Zn from the food and/or the availability of the intracellular ionic Zn (Mocchegiani *et al*, 2011). As PCs are believed to be the main cells responsible for heavy metal uptake, an imbalance in PC status during aging may be a possibility. Moreover, changes in gut microbiota have been observed upon aging (Claesson *et al*, 2011). The changes in microbiota composition might affect PCs, or the increase in PCs upon aging might be related to the changes in the microbiota. Alternatively, the intestine might be trying to keep the niche homeostasis by producing more PCs, which could lead to increased secretion of AMPs.

### Fasting and diet

**Figure 6:**
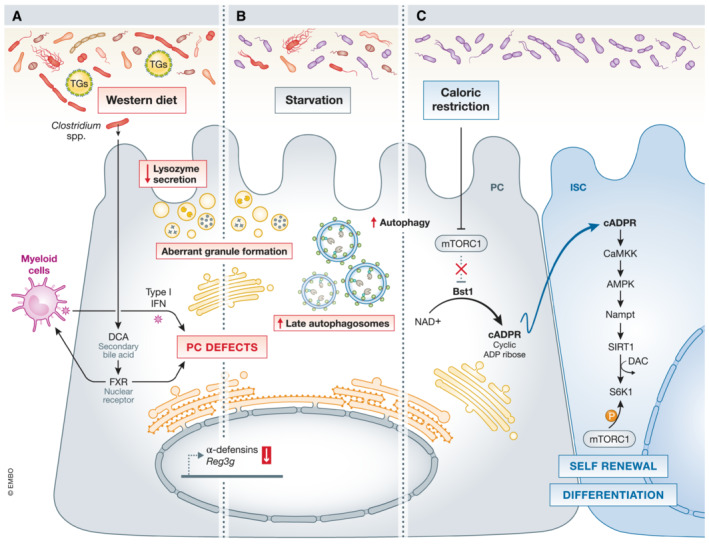
Effects of Western diet, starvation, and caloric restriction on PCs (A) The consumption of a Western diet leads to defects in PCs *via* the secondary bile acid deoxycholic acid (DCA), which is converted from cholic acid (CA) by commensal *Clostridium* spp. This increase in DCA enhances farnesoid X receptor (FXR) signaling, which also modulates the release of type I IFN by myeloid cells. Both FXR and type I IFN pathways are essential in triggering PC defects (B) Starvation has been linked to an increase in autophagy, with abundant late autophagosomes and PC defects, decreased AMP expression, and formation of aberrant granules. (C) Caloric restriction promotes ISC renewal and proliferation by inhibiting mTORC1 in PCs. mTORC1 repression enhances Bst1 and the conversion of NAD+ in cyclic ADP ribose (cADPR), which activates the AMPK/Nampt/SIRT1 pathway in ISCs. The activation of SIRT1 triggers the deacetylation of S6K1, which is further phosphorylated by mTORC1. These events are responsible for self‐renewal and differentiation in the intestinal crypts. Black arrows – pathways that are active in PCs, red arrows – “up‐ or downregulation” of factors that negatively affect PCs function.

Environmental factors such as diet can also trigger PC dysfunction or increase PC activity. In recent years, multiple studies have covered the crosstalk between diet, commensals, ingested bacteria and their byproducts, and their effect on the immune system. Diet can affect the function and number of PCs either directly or indirectly through the microbiota. The impact of diet (high‐fat diet, Western diet, Zn deficiency, alcohol consumption, or others) or starvation on the status of PCs is summarized in Table 4 and Fig 6A–C.

**Table 4:** The impact of diet on PCs and gastrointestinal function.

Diet	Effect on Paneth cells	Outcome of the diet	Factors reinforcing the effect of the diet on Paneth cells	References
Alcohol	Reduced expression of antimicrobial peptides (α‐defensins) Decreased density and size of Paneth cell granules	Increased bacterial translocation towards the liver Reduced antimicrobial activity of crypts Dysbiosis Outcome reversed by synthetic HD5 Treatment	MMP7 KO mice (α‐defensin‐deficient mice) Zinc deficiency	Purohit *et al* (2008), Zhong *et al* (2020)
Western diet	Paneth cell dysfunction	Reduced intestinal barrier function Dysbiosis		Liu *et al* (2021)
High‐fat diet	Reduced expression of antimicrobial peptides (α‐defensins and RegIII*γ*) Increased Paneth cell death	Impaired intestinal barrier function Dysbiosis	Vitamin D receptor KO mice	Su *et al* (2016), Guo *et al* (2017), Lee *et al* (2017)
Oxidized n‐3 polyunsaturated fatty acids (n3‐PUFA)	Decreased Paneth cell numbers in the duodenum compared to unoxidized n3‐PUFA	Oxidative stress and inflammation in the upper intestine		Awada *et al* (2012)
Arginine supplementation	Increased expression and secretion of AMPs	Boost of innate immune response in the small intestine		Ren *et al* (2014)
Ketogenic diet	Increased Paneth cell numbers and activity	Stimulates differentiation in the small intestine via 3‐hydroxy‐3‐methylglutaryl‐CoA synthase 2 (HMGCS2)/and the ketone body β‐hydroxybutyrate (βHB)		Wang *et al* (2017b)
Caloric restriction	Decreased mTORC1 activity in Paneth cells	Paneth cell produced cyclic ADP ribose promotes self‐renewal of intestinal stem cells		Yilmaz *et al* (2012)
Starvation	Decreased expression and secretion of antimicrobial peptides (α‐defensins, Lysozyme and RegIII*γ*) Aberrant granule formation Increased autophagy	Increased intestinal permeability Increased bacterial translocation towards mesenteric lymph nodes		Hodin *et al* (2011a)

High‐fat diet induced PC defects aggravates DSS‐induced colitis. Moreover, the PC defects induced by high‐fat diet/Western diet can be reverted by the inclusion of vitamin D, inulin, sodium butyrate, or the apoA‐I mimetic peptide 6F (Tg6F), by increasing the expression of α‐defensins, or by restoring intestinal tight junctions (Su *et al*, 2016; Beisner *et al*, 2021; Mukherjee *et al*, 2022). Consumption of antibiotics or unhealthy lifestyles, also lead to changes in microbiota and PCs. Treating mice with certain antibiotics leads to decreased expression of typical PC markers (such as *Lyz1*, *Reg3γ*, and *Defa5*), suggesting that gut microbes are important modulators of PC function and should be considered when placing patients on antibiotics for a long time. Moreover, in CD patients, the combination of tobacco smoking and mutations in *Atg16l1* gene triggers PC defects and apoptosis (Liu *et al*, 2018). As mentioned above, PCs are considered Zn‐dependent and several studies have linked Zn deficiency and genetic deficiencies of Zn transporters with PC abnormalities (Podany *et al*, 2016). Also, Adding ZnSO_4_ to the drinking water for a week protects PCs against the damage caused by TNF (Souffriau *et al*, 2020).

Diet can influence the health of PCs, and an imbalanced or inappropriate diet can lead to dysbiosis or aggravation of intestinal diseases such as CD and experimental DSS. Further research on how diet can influence PCs is needed.

## PC tools

The small number of PCs and their location in the crypts make it difficult to study them. The development of new tools, including PC‐specific mouse lines, single‐cell RNA sequencing, and PC purification and development of organoid systems have expanded our knowledge in PC biology.

### PC‐specific transgenic tools

Several gene promoters are active exclusively in PCs. Such promoters are ideal for expressing or knocking out genes of interest in PCs *via* transgenic techniques. The most important features of such promoters are that they should be specific for PCs (no ectopic expression) and allow sufficient expression of the gene of interest.

The gene of interest is cloned behind a PC‐specific promoter (several such promoters have been used), and the construct is injected in zygotes of mice. The mice are then selected for integration and PC‐specific expression. Since pronucleus injection in zygotes leads to random integration of the construct, the PC‐specificity of the promoter might be less apparent. This method was used by Garabedian *et al* (1997) to deplete PCs for the first time in 1997, by using a mouse *Defa2* promoter and a Diphtheria toxin A (DTA) gene (CR2‐tox176) construct. Overall, PCs were reduced by 82% (Garabedian *et al*, 1997; Sato *et al*, 2011a). Cell ablation using such genetic tools is elegant and more specific than the older dithizone injection technique (Sawada *et al*, 1991) and it is also more persistent (Lueschow *et al*, 2018). In 2013, a Nature paper described a PC‐specific cre line generated with a *Defa6*‐promoter‐driven cre construct (Adolph *et al*, 2013). Similarly, a *Defa4*‐promotor‐driven cre construct has been generated (Burger *et al*, 2018). Another PC cre line was created via homologous recombination of CreER in the *Lyz1* locus of embryonic stem cells (Van Es *et al*, 2019). Crossing a PC‐specific cre mouse with a reporter mouse, a mouse with floxed alleles, or a floxed‐STOP DTA receptor mouse, produces mice with colored PCs, PC‐specific KO of the floxed alleles, or mice with PC depletion after the injection of DTA, respectively (Shankman *et al*, 2021).

Tools to increase the number of PCs have also been developed. Hayase *et al* (2017) demonstrated that injection of mice with R‐spondin1, a growth and differentiation factor of PCs, led to crypt hyperproliferation and more than double the number of PCs per crypt (Hayase *et al*, 2017). The concentrations of DEFA1 and DEFA4 protein in the mouse feces was increased, and hence these two peptides might be considered as PC biomarkers. Moreover, oral supplementation of mice with certain probiotic strains, such as *Lactobacillus casei* and *Lactococcus paracasei*, led to increases in PC numbers in mouse crypts associated with increased antimicrobial activity against pathogens (Cazorla *et al*, 2018).

### Single‐cell sequencing data of PCs

Single‐cell RNA sequencing (scRNA‐seq) on the small intestine of mice and humans gave considerable information about cellular diversity and interesting insights in PCs and other intestinal cell types (Grün *et al*, 2015; Haber *et al*, 2018; Wang *et al*, 2020). Grün *et al* (2015) performed scRNA‐seq on organoids of mice and developed an algorithm (RaceID) to identify rare cell types in complex populations (Grün *et al*, 2015). Haber *et al*, 2018 were the first to perform scRNA‐seq on cells extracted from the small intestine of mice and listed the signature genes for each identified IEC type. For PCs, they identified 82 signature genes, 12 of them being α‐defensins, three cryptdin‐related sequence peptide‐coding genes, and some known PC markers (e.g. *Lyz1*). Haber *et al*, 2018 identified in this way a new PC‐marker, *Mptx2*, which colocalized *via* ISH with LYZ in the PCs, and nowhere else in the small intestine. Furthermore, they identified 2 PC subtypes in the small intestine. These two populations represent regional variation as all the marker genes from the first subtype were enriched in PCs of the ileum, while 70% of the PC marker genes of the second subtype were enriched in duodenum and jejunum. The first subtype expressing more α‐defensins, suggesting that PCs located in the ileum are more antimicrobial active. Single cell data on sorted PCs could learn us more about the intrinsic variability of these cells, for example, identifying heterogeneous PC populations or PCs in a different differentiation status, as was performed on sorted mucin2 (MUC2)‐positive goblet cells with the use of a MUC2 reporter mouse line (Nyström *et al*, 2021). In this study several distinct goblet cell populations (after the removal of MUC2‐expressing PCs), correlating with different functions, were identified. The authors described a subpopulation of goblet cells, with a clear microbial defense profile (e.g., antimicrobial genes), suggesting at least a partial overlapping gene signature between goblet cells and PCs (Nyström *et al*, 2021).

In 2020, a scRNA‐seq experiment was performed on three compartments of the human gastrointestinal tract, ileum, colon, and rectum (Wang *et al*, 2020), which shed more light on the differential nutrient absorption functions in the different compartments. Moreover, the authors identified Paneth‐like cells in the large intestine that shared a group of highly expressed genes with PCs. These genes encode some AMPs (e.g., *LYZ*), genes that serve as niche factors for the ISCs (e.g., *EGF*, *WNT3A*, and *Notch*) and transcription factors involved in differentiation. However, Paneth‐like cells in the large intestine, unlike PCs, do not express *HD5*, *HD6*, *REG1A*, and *REG3A*, suggesting a more prominent role of PCs in microbial defense. However, another recent scRNAseq performed on different compartments of the human gut shows that *LYZ* expression is not unique to PCs in the gut, and that PCs are devoid of some niche factors for ISCs (e.g., *EGF* and *WNT3A*) (Burclaff *et al*, 2022).

### Purification of PCs and organoids

Before 2009, PC studies were practically limited to *in vivo* studies (e.g., antimicrobial activity assays, microscopy, and qPCR on PC markers) as no culture system was available. Clevers *et al* published in 2009 a new 3D *in vitro* culture system containing a mix of all the differentiated IECs found in the small intestine, named mini‐gut organoids or enteroids (Sato *et al*, 2009). A major advantage of such models is that specific growth factors can be added to skew the differentiation to the desired lineage. Differentiation of organoids can be forced towards PCs by adding a notch inhibitor (DAPT) and an inhibitor of GSK3β‐mediated β‐catenin degradation (CHIR99021) (Treveil *et al*, 2020). This is an elegant model to study PCs in the context of other epithelial cells, but it is not possible to study direct effects exclusively on PCs. A 2D monolayer culture method starting from porcine organoids has also been described. It consists of granular PCs and other cell types. This method has the advantage that the apical surface is easily accessible, and that it has high TEER values (1000 Ω/cm^2^), making it of interest for testing the effect of drugs and nutrients on epithelial membrane integrity (van der Hee *et al*, 2018).

A protocol to purify PCs was initially described by Clevers' group (Sato *et al*, 2011a) and further optimized (Schmitt *et al*, 2018). Briefly, crypts are purified from the small intestine and dissociated to obtain a single‐cell suspension. Single cells are then stained for two PC makers, CD24 and C‐kit, and hematopoietic lineage antibodies (CD31, CD45, and Ter119). PCs are gated on CD24^+^C‐kit^+^SCC^Medium‐High^ CD31^−^CD45^−^TER119^−^ cells and sorted. The sorted PCs can be kept in culture for a short time or used to perform RNAseq (Yu *et al*, 2018). Another way to study or sort PCs is by using PC‐specific fluorescent reporter lines.

Laser capture dissection is another method that can be used to isolate a region tissue of interest (e.g., PCs from small intestinal cryosections) with a laser beam. This method enables the study of RNA expression in PCs (Vaishnava *et al*, 2008). There is still no (immortalized) PC line commercially available, hampering *in vitro* studies on pure PCs.

## Conclusions

Paneth cells play a key role in preserving gut homeostasis. Their decisive functions include (i) providing niche factors to the ISC compartment, (ii) regulating the composition of the microbiome by producing AMPs, (iii) dampening inflammation by phagocytosis and efferocytosis, (iv) uptake of heavy metals, and (v) preserving barrier integrity. Dysfunction of PCs can disturb one or more of these functions, leading to an imbalance in the gastrointestinal tract. Decreased or dysfunctional PCs, followed by escape of bacteria towards different organs, is often observed in several diseases, such as infectious diseases, IBD (CD and ulcerative colitis) and GVHD. Genetic disorders or environmental triggers can cause ERS or autophagy deficits in PCs, which are clearly linked with intestinal diseases. The challenges in PC studies relate to the location of PCs and their limited number. In particular, the lack of a pure PC line to study direct effects is a major impediment. Until recently, PC expression studies have relied on ISH or the use of PC‐specific markers on ileum biopsies. Fortunately, PCs can now be sorted to study the expression signature of pure PCs in different (disease) models. Moreover, the PC‐specific cre tool has recently been used to confirm earlier PC results obtained in villin^KO^ models. The recent development of new tools and techniques has led to a more rapid expansion of knowledge on PCs.

**Pending issues**

- Genetic disorders, environmental factors, and diet have been linked to Paneth cell dysfunctions in mice. However, these factors have not been adequately studied in human samples or biopsies.
- Studying Paneth cells in human patients is problematic because it requires invasive biopsies. A good biomarker that represents the health state of Paneth cells and can be measured in body fluids (urine or blood) or feces would therefore be of value.
- The challenges in Paneth cell studies relate to their limited number and deep location. Establishing a pure Paneth cell‐line would make it possible to directly study the effects on Paneth cells and to use them as an assay for screening compounds.
- Different inbred mouse strains under identical housing conditions differ in the number of Paneth cells, the expression of antimicrobial peptides and in α‐defensin genes and proteins. Moreover, these strains are kept in captivity over many decades, making them vulnerable to genetic drift. To better mimic the human situation, Paneth cell studies on wild mice or wildling mice might be more relevant, but they are also more complex.

## Author contributions

**Charlotte Wallaeys:** Writing—original draft; writing—review and editing; figures and tables—design and original draft, figures and tables—review and editing. **Natalia Garcia Gonzalez:** Writing—original draft; writing—review and editing; figures and tables—design and original draft, figures and tables—review and editing. **Claude Libert:** Writing—original draft; writing—review and editing.

## Disclosure and competing interests statement

Prof. Claude Libert is a member of the EMBO Molecular Medicine Editorial Board. This has no bearing on the editorial consideration of this article for publication. The other authors declare that they have no conflict of interest.

## References

[emmm202216427-bib-0001] Adolph TE , Tomczak MF , Niederreiter L , Ko HJ , Böck J , Martinez‐Naves E , Glickman JN , Tschurtschenthaler M , Hartwig J , Hosomi S *et al* (2013) Paneth cells as a site of origin for intestinal inflammation. Nature 503: 272–276 24089213 10.1038/nature12599 PMC3862182

[emmm202216427-bib-0002] Aley SB , Zimmerman M , Hetsko M , Selsted ME , Gillin FD (1994) Killing of *Giardia lamblia* by cryptdins and cationic neutrophil peptidest. Infect Immun 62: 5397–5403 7960119 10.1128/iai.62.12.5397-5403.1994 PMC303280

[emmm202216427-bib-0003] Ali A , Tan HY , Kaiko GE (2020) Role of the intestinal epithelium and its interaction with the microbiota in food allergy. Front Immunol 11: 604054 33365031 10.3389/fimmu.2020.604054 PMC7750388

[emmm202216427-bib-0004] Almohazey D , Lo YH , Vossler CV , Simmons AJ , Hsieh JJ , Bucar EB , Schumacher MA , Hamilton KE , Lau KS , Shroyer NF *et al* (2017) The ErbB3 receptor tyrosine kinase negatively regulates Paneth cells by PI3K‐dependent suppression of Atoh1. Cell Death Differ 24: 855–865 28304405 10.1038/cdd.2017.27 PMC5423110

[emmm202216427-bib-0005] Ara T , Hashimoto D (2021) Novel insights into the mechanism of GVHD‐induced tissue damage. Front Immunol 12: 713631 34512636 10.3389/fimmu.2021.713631 PMC8429834

[emmm202216427-bib-0006] Araujo A , Safronova A , Burger E , López‐Yglesias A , Giri S , Camanzo ET , Martin AT , Grivennikov SI , Yarovinsky F (2021) IFN‐γ mediates paneth cell death via suppression of mtor. eLife 10: e60478 34633285 10.7554/eLife.60478 PMC8570691

[emmm202216427-bib-0007] Awada M , Soulage CO , Meynier A , Debard C , Plaisancié P , Benoit B , Picard G , Loizon E , Chauvin M , Estienne M *et al* (2012) Dietary oxidized n‐3 PUFA induce oxidative stress and infl ammation: role of intestinal absorption of 4‐HHE and reactivity in intestinal cells. J Lipid Res 53: 2069–2080 22865918 10.1194/jlr.M026179 PMC3435540

[emmm202216427-bib-0008] Ayabe T , Satchell DP , Wilson CL , Parks WC , Selsted ME , Ouellette AJ (2000) Secretion of microbicidal α‐defensins by intestinal Paneth cells in response to bacteria. Nat Immunol 1: 113–118 11248802 10.1038/77783

[emmm202216427-bib-0009] Ballegeer M , Van LK , Timmermans S , Eggermont M , Vandevyver S , Thery F , Dendoncker K , Souffriau J , Vandewalle J , Van WL *et al* (2018) Glucocorticoid receptor dimers control intestinal STAT1 and TNF‐induced inflammation in mice. J Clin Invest 128: 3265–3279 29746256 10.1172/JCI96636 PMC6063488

[emmm202216427-bib-0010] Barker N , Van Oudenaarden A , Clevers H (2012) Perspective identifying the stem cell of the intestinal Crypt: strategies and pitfalls. Stem Cell 11: 452–460 10.1016/j.stem.2012.09.009 23040474

[emmm202216427-bib-0011] Barreto E , Barreto L , Rattes IC , da Costa AV , Gama P (2022) Paneth cells and their multiple functions. Cell Biol Int 46: 701–710 35032139 10.1002/cbin.11764

[emmm202216427-bib-0012] Bastide P , Darido C , Pannequin J , Kist R , Robine S , Marty‐Double C , Bibeau F , Scherer G , Joubert D , Hollande F *et al* (2007) Sox9 regulates cell proliferation and is required for Paneth cell differentiation in the intestinal epithelium. J Cell Biol 178: 635–648 17698607 10.1083/jcb.200704152 PMC2064470

[emmm202216427-bib-0013] Beisner J , Filipe Rosa L , Kaden‐Volynets V , Stolzer I , Günther C , Bischoff SC (2021) Prebiotic inulin and sodium butyrate attenuate obesity‐induced intestinal barrier dysfunction by induction of antimicrobial peptides. Front Immunol 12: 678360 34177920 10.3389/fimmu.2021.678360 PMC8226265

[emmm202216427-bib-0014] Bel S , Pendse M , Wang Y , Li Y , Ruhn KA , Hassell B , Leal T , Winter SE , Xavier RJ , Hooper LV (2017) Paneth cells secrete lysozyme via secretory autophagy during bacterial infection of the intestine. Science 1052: 1047–1052 10.1126/science.aal4677 PMC5702267 28751470

[emmm202216427-bib-0015] Bevins CL , Salzman NH (2011) Paneth cells, antimicrobial peptides and maintenance of intestinal homeostasis. Nat Rev Microbiol 9: 356–368 21423246 10.1038/nrmicro2546

[emmm202216427-bib-0016] Bjerknes M , Cheng H (2010) Cell Lineage metastability in Gfi1‐deficient mouse intestinal epithelium. Dev Biol 345: 49–63 20599897 10.1016/j.ydbio.2010.06.021

[emmm202216427-bib-0017] Burclaff J , Bliton RJ , Breau KA , Ok MT , Gomez‐Martinez I , Ranek JS , Bhatt AP , Purvis JE , Woosley JT , Magness ST (2022) A proximal‐to‐distal survey of healthy adult human small intestine and colon epithelium by single‐cell transcriptomics. Cell Mol Gastroenterol Hepatol 13: 1554–1589 35176508 10.1016/j.jcmgh.2022.02.007 PMC9043569

[emmm202216427-bib-0018] Burger E , Araujo A , López‐Yglesias A , Rajala MW , Geng L , Levine B , Hooper LV , Burstein E , Yarovinsky F (2018) Loss of paneth cell autophagy causes acute susceptibility to toxoplasma gondii‐mediated inflammation. Cell Host Microbe 23: 177–190.e4 29358083 10.1016/j.chom.2018.01.001 PMC6179445

[emmm202216427-bib-0019] Cabrera S , Fernández ÁF , Mariño G , Aguirre A , Suárez MF , Español Y , Vega JA , Laurà R , Fueyo A , Soledad Fernández‐García M *et al* (2013) ATG4B/autophagin‐1 regulates intestinal homeostasis and protects mice from experimental colitis. Autophagy 9: 1188–1200 23782979 10.4161/auto.24797 PMC3748191

[emmm202216427-bib-0020] Cadwell K , Liu J , Brown SL , Miyoshi H , Loh J , Lennerz J , Kishi C , Kc W , Carrero J a , Hunt S *et al* (2008) A unique role for autophagy and Atg16L1 in Paneth cells in murine and human intestine. Nature 456: 259–263 18849966 10.1038/nature07416 PMC2695978

[emmm202216427-bib-0021] Cadwell K , Patel KK , Komatsu M , Virgin HW IV , Stappenbeck TS (2009) A common role for Atg16L1, Atg5 and Atg7 in small intestinal Paneth cells and Crohn disease. Autophagy 5: 250–252 19139628 10.4161/auto.5.2.7560 PMC2940227

[emmm202216427-bib-0022] Cazorla SI , Maldonado‐Galdeano C , Weill R , De Paula J , Perdigón GDV (2018) Oral administration of probiotics increases Paneth cells and intestinal antimicrobial activity. Front Microbiol 9: 1–14 29713315 10.3389/fmicb.2018.00736 PMC5911494

[emmm202216427-bib-0023] Chakraborti S , Chatterjee T , Joshi P , Poddar A , Bhattacharyya B , Singh SP , Gupta V , Chakrabarti P (2010) Structure and activity of lysozyme on binding to ZnO nanoparticles. Langmuir 26: 3506–3513 20000758 10.1021/la903118c

[emmm202216427-bib-0024] Cheng H , Leblond CP (1974) Origin, differentiation and renewal of the four main epithelial cell types in the mouse small intestine I. Columnar cell. Am J Anat 141: 461–479 4440632 10.1002/aja.1001410403

[emmm202216427-bib-0025] Chiang HY , Lu HH , Sudhakar JN , Chen YW , Shih NS , Weng YT , Shui JW (2022) IL‐22 initiates an IL‐18‐dependent epithelial response circuit to enforce intestinal host defence. Nat Commun 13: 874 35169117 10.1038/s41467-022-28478-3 PMC8847568

[emmm202216427-bib-0026] Chu H , Pazgier M , Jung G , Nuccio SP , Castillo PA , De Jong MF , Winter MG , Winter SE , Wehkamp J , Shen B *et al* (2012) Human α‐defensin 6 promotes mucosal innate immunity through self‐assembled peptide nanonets. Science 337: 477–481 22722251 10.1126/science.1218831 PMC4332406

[emmm202216427-bib-0027] Claesson MJ , Cusack S , O'Sullivan O , Greene‐Diniz R , De Weerd H , Flannery E , Marchesi JR , Falush D , Dinan T , Fitzgerald G *et al* (2011) Composition, variability, and temporal stability of the intestinal microbiota of the elderly. Proc Natl Acad Sci USA 108: 4586–4591 20571116 10.1073/pnas.1000097107 PMC3063589

[emmm202216427-bib-0028] Cray P , Sheahan BJ , Dekaney CM (2021) Secretory sorcery: paneth cell control of intestinal repair and homeostasis. Cell Mol Gastroenterol Hepatol 12: 1239–1250 34153524 10.1016/j.jcmgh.2021.06.006 PMC8446800

[emmm202216427-bib-0029] Croguennec T , Nau F , Molle D , Le Graet Y , Brule G (2000) Iron and citrate interactions with hen egg white lysozyme. Food Chem 68: 29–35

[emmm202216427-bib-0030] Daher KA , Selsted ME , Lehrer RI (1986) Direct inactivation of viruses by human granulocyte defensins. J Virol 60: 1068–1074 3023659 10.1128/jvi.60.3.1068-1074.1986 PMC253347

[emmm202216427-bib-0031] Danscher G , Thorlacius‐ussing O , Rungby J (1985) Selenium in the Paneth cells. Sci Total Environ 42: 189–192 2409594 10.1016/0048-9697(85)90019-1

[emmm202216427-bib-0032] Das S , Yu S , Sakamori R , Vedula P , Feng Q , Flores J , Hoffman A , Fu J , Stypulkowski E , Rodriguez A *et al* (2015) Rab8a vesicles regulate Wnt ligand delivery and Paneth cell maturation at the intestinal stem cell niche. Development 142: 2147–2162 26015543 10.1242/dev.121046 PMC4483769

[emmm202216427-bib-0033] Decker T , Müller M , Stockinger S (2005) The Yin and Yang of type I interferon activity in bacterial infection. Nat Rev Immunol 5: 675–687 16110316 10.1038/nri1684

[emmm202216427-bib-0034] Derde M , Lechevalier V , Guérin‐Dubiard C , Cochet MF , Jan S , Baron F , Gautier M , Vié V , Nau F (2013) Hen egg white lysozyme permeabilizes Escherichia coli outer and inner membranes. J Agric Food Chem 61: 9922–9929 24047287 10.1021/jf4029199

[emmm202216427-bib-0035] Dessein R , Gironella M , Vignal C , Peyrin‐Biroulet L , Sokol H , Secher T , Lacas‐Gervais S , Gratadoux JJ , Lafont F , Dagorn JC *et al* (2009) Toll‐like receptor 2 is critical for induction of Reg3b expression and intestinal clearance of Yersinia pseudotuberculosis. Gut 58: 771–776 19174417 10.1136/gut.2008.168443

[emmm202216427-bib-0036] Deuring JJ , Fuhler GM , Konstantinov SR , Peppelenbosch MP , Kuipers EJ , De Haar C , Van Der Woude CJ (2014) Genomic ATG16L1 risk allele‐restricted Paneth cell ER stress in quiescent Crohn's disease. Gut 63: 1081–1091 23964099 10.1136/gutjnl-2012-303527

[emmm202216427-bib-0037] Durand A , Donahue B , Peignon G , Letourneur F , Cagnard N , Slomianny C , Perret C , Shroyer NF , Romagnolo B (2012) Functional intestinal stem cells after Paneth cell ablation induced by the loss of transcription factor Math1 (Atoh1). Proc Natl Acad Sci USA 109: 8965–8970 22586121 10.1073/pnas.1201652109 PMC3384132

[emmm202216427-bib-0038] Eriguchi Y , Nakamura K , Yokoi Y , Sugimoto R , Takahashi S , Hashimoto D , Teshima T , Ayabe T , Selsted ME , Ouellette AJ (2018) Essential role of IFN‐γ in T cell‐associated intestinal inflammation. JCI Insight 3: 1–18 10.1172/jci.insight.121886 PMC6237234 30232288

[emmm202216427-bib-0039] Erlandsen S , Chase D (1972) Paneth cell function: phagocytosis and intracellular digestion of intestinal microorganisms: spiral microorganisms. Ultrastruct Res 41: 319–333 10.1016/s0022-5320(72)90072-x 4636022

[emmm202216427-bib-0040] van Es JH , Jay P , Gregorieff A , van Gijn ME , Jonkheer S , Hatzis P , Thiele A , van den Born M , Begthel H , Brabletz T *et al* (2005) Wnt signalling induces maturation of Paneth cells in intestinal crypts. Nat Cell Biol 7: 381–386 15778706 10.1038/ncb1240

[emmm202216427-bib-0041] Farin HF , Karthaus WR , Kujala P , Rakhshandehroo M , Schwank G , Vries RGJ , Kalkhoven E , Nieuwenhuis EES , Clevers H (2014) Paneth cell extrusion and release of antimicrobial products is directly controlled by immune cell‐derived IFN‐γ. J Exp Med 211: 1393–1405 24980747 10.1084/jem.20130753 PMC4076587

[emmm202216427-bib-0042] Fernandes PR , Samuelson DA , Clark WR , Cousins RJ (1997) Immunohistochemical localization of cysteine‐rich intestinal protein in rat small intestine. Am J Physiol 272: 751–759 10.1152/ajpgi.1997.272.4.G751 9142905

[emmm202216427-bib-0043] Garabedian EM , Roberts LJJ , McNevin MS , Gordon JI (1997) Examining the role of Paneth cells in the small intestine by lineage ablation in transgenic mice. J Biol Chem 272: 23729–23740 9295317 10.1074/jbc.272.38.23729

[emmm202216427-bib-0044] Gassler N (2017) Paneth cells in intestinal physiology and pathophysiology. World J Gastrointest Pathophysiol 8: 150–160 29184701 10.4291/wjgp.v8.i4.150 PMC5696613

[emmm202216427-bib-0045] Gaudino SJ , Beaupre M , Lin X , Joshi P , Rathi S , McLaughlin PA , Kempen C , Mehta N , Eskiocak O , Yueh B *et al* (2021) IL‐22 receptor signaling in Paneth cells is critical for their maturation, microbiota colonization, Th17‐related immune responses, and anti‐Salmonella immunity. Mucosal Immunol 14: 389–401 33060802 10.1038/s41385-020-00348-5 PMC7946635

[emmm202216427-bib-0046] Geiser J , Venken KJT , de Lisle RC , Andrews GK (2012) A mouse model of acrodermatitis enteropathica: Loss of intestine zinc transporter ZIP4 (Slc39a4) disrupts the stem cell niche and intestine integrity. PLoS Genet 8: e1002766 22737083 10.1371/journal.pgen.1002766 PMC3380849

[emmm202216427-bib-0047] Ghosh D , Porter E , Shen B , Lee SK , Wilk D , Drazba J , Yadav SP , Crabb JW , Ganz T , Bevins CL (2002) Paneth cell trypsin is the processing enzyme for human defensin‐5. Nat Immunol 3: 583–590 12021776 10.1038/ni797

[emmm202216427-bib-0048] Giblin LJ , Chang CJ , Bentley AF , Frederickson C , Lippard SJ , Frederickson CJ (2006) Zinc‐secreting Paneth cells studied by ZP fluorescence. J Histochem Cytochem 54: 311–316 16260591 10.1369/jhc.5A6724.2005

[emmm202216427-bib-0049] Goga A , Yagabasan B , Herrmanns K , Godbersen S , Silva PN , Denzler R , Zünd M , Furter M , Schwank G , Sunagawa S *et al* (2021) miR‐802 regulates Paneth cell function and enterocyte differentiation in the mouse small intestine. Nat Commun 12: 3339 34099655 10.1038/s41467-021-23298-3 PMC8184787

[emmm202216427-bib-0050] González‐Navajas JM , Lee J , David M , Raz E (2012) Immunomodulatory functions of type I interferons. Nat Rev Immunol 12: 125–135 22222875 10.1038/nri3133 PMC3727154

[emmm202216427-bib-0051] Gregorieff A , Stange DE , Kujala P , Begthel H , van den Born M , Korving J , Peters PJ , Clevers H (2009) The ETS‐domain transcription factor SPDEF promotes maturation of goblet and Paneth cells in the intestinal epithelium. Gastroenterology 137: 1333–1345 19549527 10.1053/j.gastro.2009.06.044

[emmm202216427-bib-0052] Grinat J , Kosel F , Goveas N , Kranz A , Alexopoulou D , Rajewsky K , Sigal M , Stewart AF , Heuberger J (2022) Epigenetic modifier balances Mapk and Wnt signalling in differentiation of goblet and Paneth cells. Life Sci Alliance 5: 1–14 10.26508/lsa.202101187 PMC8807877 35064075

[emmm202216427-bib-0053] Grootjans J , Hodin CM , Haan JJDE , Derikx JPM (2011) Level of activation of the unfolded protein response correlates with Paneth cell apoptosis in human small intestine exposed to ischemia/reperfusion. Gastroenterology 140: 529–539 20965186 10.1053/j.gastro.2010.10.040

[emmm202216427-bib-0054] Grün D , Lyubimova A , Kester L , Wiebrands K , Basak O , Sasaki N , Clevers H , Van Oudenaarden A (2015) Single‐cell messenger RNA sequencing reveals rare intestinal cell types. Nature 525: 251–255 26287467 10.1038/nature14966

[emmm202216427-bib-0055] Gulati AS , Shanahan MT , Arthur JC , Grossniklaus E , von Furstenberg RJ , Kreuk L , Henning SJ , Jobin C , Sartor RB (2012) Mouse background strain profoundly influences paneth cell function and intestinal microbial composition. PLoS One 7: e32403 22384242 10.1371/journal.pone.0032403 PMC3288091

[emmm202216427-bib-0056] Günther C , Martini E , Wittkopf N , Amann K , Weigmann B , Neumann H , Waldner M , Hedrick SM , Tenzer S , Markus F *et al* (2012) Caspase‐8 regulates TNF‐a‐induced epithelial necroptosis and terminal ileitis. Nature 477: 335–339 10.1038/nature10400 PMC3373730 21921917

[emmm202216427-bib-0057] Günther C , Ruder B , Stolzer I , Dorner H , He GW , Chiriac MT , Aden K , Strigli A , Bittel M , Zeissig S *et al* (2019) Interferon lambda promotes Paneth cell death via STAT1 signaling in mice and is increased in inflamed ileal tissues of patients with Crohn's disease. Gastroenterology 157: 1310–1322.e13 31352002 10.1053/j.gastro.2019.07.031

[emmm202216427-bib-0058] Guo X , Li J , Tang R , Zhang G , Zeng H , Wood RJ , Liu Z (2017) High fat diet alters gut microbiota and the expression of Paneth cell‐antimicrobial peptides preceding changes of circulating inflammatory cytokines. Mediators Inflamm 2017: 9474896 28316379 10.1155/2017/9474896 PMC5339499

[emmm202216427-bib-0059] Gyongyosi B , Cho Y , Lowe P , Calenda CD , Iracheta‐Vellve A , Satishchandran A , Ambade A , Szabo G (2019) Alcohol‐induced IL‐17A production in Paneth cells amplifies endoplasmic reticulum stress, apoptosis, and inflammasome‐IL‐18 activation in the proximal small intestine in mice. Mucosal Immunol 12: 930–944 31105269 10.1038/s41385-019-0170-4 PMC6599481

[emmm202216427-bib-0060] Haber AL , Biton M , Rogel N , Herbst RH , Smillie C , Burgin G , Delorey TM , Howitt MR , Katz Y , Tirosh I *et al* (2018) A single‐cell survey of the small intestinal epithelium Adam. Nature 551: 333–339 10.1038/nature24489 PMC6022292 29144463

[emmm202216427-bib-0061] Hadjicharalambous C , Sheynis T , Jelinek R , Shanahan MT , Ouellette AJ , Gizeli E (2005) Mechanisms of α‐defensin bactericidal action. Biochemistry 23: 1–7 10.1021/bi800335e PMC2645939 18973303

[emmm202216427-bib-0062] Hanash AM , Dudakov JA , Hua G , Connor MHO , Lauren F , Singer NV , West ML , Jenq RR , Holland AM , Kappel W *et al* (2013) Interleukin‐22 protects intestinal stem cells from immune‐mediated tissue damage and regulates sensitivity to graft versus host disease. Immunity 37: 339–350 10.1016/j.immuni.2012.05.028 PMC3477611 22921121

[emmm202216427-bib-0063] Hayase E , Hashimoto D , Nakamura K , Noizat C , Ogasawara R , Takahashi S , Ohigashi H , Yokoi Y , Sugimoto R , Matsuoka S *et al* (2017) R‐Spondin1 expands Paneth cells and prevents dysbiosis induced by graft‐versus‐host disease. J Exp Med 214: 3507–3518 29066578 10.1084/jem.20170418 PMC5716036

[emmm202216427-bib-0064] van der Hee B , Loonen LMP , Taverne N , Taverne‐Thiele JJ , Smidt H , Wells JM (2018) Optimized procedures for generating an enhanced, near physiological 2D culture system from porcine intestinal organoids. Stem Cell Res 28: 165–171 29499500 10.1016/j.scr.2018.02.013

[emmm202216427-bib-0065] Heuberger J , Kosel F , Qi J , Grossmann KS , Rajewsky K , Birchmeier W (2014) Shp2 / MAPK signaling controls goblet/paneth cell fate decisions in the intestine. Proc Natl Acad Sci U S A 111: 3472–3477 24550486 10.1073/pnas.1309342111 PMC3948231

[emmm202216427-bib-0066] Hirao LA , Grishina I , Bourry O , Hu WK , Somrit M , Sankaran‐Walters S , Gaulke CA , Fenton AN , Li JA , Crawford RW *et al* (2014) Early mucosal sensing of SIV infection by Paneth cells induces IL‐1β production and initiates gut epithelial disruption. PLoS Pathog 10: e1004311 25166758 10.1371/journal.ppat.1004311 PMC4148401

[emmm202216427-bib-0067] Hodin CM , Lenaerts K , Grootjans J , De Haan JJ , Hadfoune M , Verheyen FK , Kiyama H , Heineman E , Buurman WA (2011a) Starvation compromises Paneth cells. Am J Pathol 179: 2885–2893 21986443 10.1016/j.ajpath.2011.08.030 PMC3260859

[emmm202216427-bib-0068] Hodin CM , Verdam FJ , Grootjans J , Rensen SS , Verheyen FK , Dejong CHC , Buurman WA , Greve JW , Lenaerts K (2011b) Reduced Paneth cell antimicrobial protein levels correlate with activation of the unfolded protein response in the gut of obese individuals. J Pathol 225: 276–284 21630271 10.1002/path.2917

[emmm202216427-bib-0069] Hueber W , Sands BE , Lewitzky S , Vandemeulebroecke M , Reinisch W , Higgins PDR , Wehkamp J , Feagan BG , Yao MD , Karczewski M *et al* (2012) Secukinumab, a human anti‐IL‐17A monoclonal antibody, for moderate to severe Crohn's disease: Unexpected results of a randomised, double‐blindplacebo‐ controlled trial. Gut 61: 1693–1700 22595313 10.1136/gutjnl-2011-301668 PMC4902107

[emmm202216427-bib-0070] Ireland H , Houghton C , Howard L , Winton DJ (2005) Cellular inheritance of a Cre‐activated reporter gene to determine Paneth cell longevity in the murine small intestine. Dev Dyn 233: 1332–1336 15937933 10.1002/dvdy.20446

[emmm202216427-bib-0071] Ishimoto YM , Shono Y , Gomez LE , Lucey VMH , Cammer M , Neil J , Dewan MZ , Lieberman SR , Lazrak A , Marinis JM *et al* (2017) Autophagy protein ATG16L1 prevents necroptosis in the intestinal epithelium. J Exp Med 214: 3687–3705 29089374 10.1084/jem.20170558 PMC5716041

[emmm202216427-bib-0072] Jones EJ , Matthews ZJ , Gul L , Sudhakar P , Treveil A , Divekar D , Buck J , Wrzesinski T , Jefferson M , Armstrong SD *et al* (2019) Integrative analysis of Paneth cell proteomic and transcriptomic data from intestinal organoids reveals functional processes dependent on autophagy. Dis Model Mech 12: dmm037069 30814064 10.1242/dmm.037069 PMC6451430

[emmm202216427-bib-0073] Kai‐Larsen Y , Bergsson G , Gudmundsson GH , Printz G , JöRnvall H , Marchini G , Agerberth B (2007) Antimicrobial components of the neonatal gut affected upon colonization. Pediatr Res 61: 530–536 17413858 10.1203/pdr.0b013e318045be83

[emmm202216427-bib-0074] Kaser A , Lee A , Franke A , Glickman JN , Zeissig S , Tilg H , Nieuwenhuis EES , Higgins DE , Schreiber S , Glimcher LH *et al* (2008) XBP1 links ER stress to intestinal inflammation and confers genetic risk for human inflammatory bowel disease. Cell 134: 743–756 18775308 10.1016/j.cell.2008.07.021 PMC2586148

[emmm202216427-bib-0075] Khaloian S , Rath E , Hammoudi N , Gleisinger E , Blutke A , Giesbertz P , Berger E , Metwaly A , Waldschmitt N , Allez M *et al* (2020) Mitochondrial impairment drives intestinal stem cell transition into dysfunctional Paneth cells predicting Crohn's disease recurrence. Gut 69: 1939–1951 32111634 10.1136/gutjnl-2019-319514 PMC7569388

[emmm202216427-bib-0076] Kip AM , Ceulemans LJ , Hundscheid IHR , Canovai E , Hartog H , Brown RM , Corcos O , Joly F , De Hertogh G , Gupte G *et al* (2020) Paneth cell alterations during ischemia‐reperfusion, follow‐up, and graft rejection after intestinal transplantation. Transplantation 104: 1952–1958 32265415 10.1097/TP.0000000000003257

[emmm202216427-bib-0077] Kobayashi KS , Chamaillard M , Ogura Y , Henegariu O , Inohara N , Nuñez G , Flavell RA (2005) Nod2‐dependent regulation of innate and adaptive immunity in the intestinal tract. Science 307: 731–734 15692051 10.1126/science.1104911

[emmm202216427-bib-0078] Lassen KG , Kuballa P , Conway KL , Patel KK , Becker CE (2014) Atg16L1 T300A variant decreases selective autophagy resulting in altered cytokine signaling and decreased antibacterial defense. Proc Natl Acad Sci USA 111: 7741–7746 24821797 10.1073/pnas.1407001111 PMC4040621

[emmm202216427-bib-0079] Lee J , Lee H , Kim TK , Kim M , Park YM , Kim J , Park K , Kweon M , Kim S , Bae J *et al* (2017) Obesogenic diet‐induced gut barrier dysfunction and pathobiont expansion aggravate experimental colitis. PLoS One 12: e0187515 29107964 10.1371/journal.pone.0187515 PMC5673181

[emmm202216427-bib-0080] Lee YS , Kim TY , Kim Y , Lee SH , Kim S , Kang SW , Yang JY , Baek IJ , Sung YH , Park YY *et al* (2018) Microbiota‐derived lactate accelerates intestinal stem‐cell‐mediated epithelial development. Cell Host Microbe 24: 833–846.e6 30543778 10.1016/j.chom.2018.11.002

[emmm202216427-bib-0081] Levine JE , Huber E , Hammer STG , Harris AC , Greenson JK , Braun TM , Ferrara JLM , Holler E (2013) Low Paneth cell numbers at onset of gastrointestinal graft‐versus‐host disease identify patients at high risk for nonrelapse mortality. Blood 122: 1505–1509 23760615 10.1182/blood-2013-02-485813 PMC3750343

[emmm202216427-bib-0082] Liu SY , Sanchez DJ , Aliyari R , Lu S , Cheng G (2012) Systematic identification of type I and type II interferon‐induced antiviral factors. Proc Natl Acad Sci USA 109: 4239–4244 22371602 10.1073/pnas.1114981109 PMC3306696

[emmm202216427-bib-0083] Liu B , Gulati AS , Cantillana V , Henry SC , Schmidt EA , Daniell X , Grossniklaus E , Schoenborn AA , Sartor RB , Taylor GA (2013) Irgm1‐deficient mice exhibit paneth cell abnormalities and increased susceptibility to acute intestinal inflammation. Am J Physiol Gastrointest Liver Physiol 305: 573–584 10.1152/ajpgi.00071.2013 PMC3798734 23989005

[emmm202216427-bib-0084] Liu G , Ren W , Fang J , An C , Hu A , Guan G (2017a) l ‐ Glutamine and l ‐ arginine protect against enterotoxigenic Escherichia coli infection via intestinal innate immunity in mice. Amino Acids 49: 1945–1954 28299479 10.1007/s00726-017-2410-9

[emmm202216427-bib-0085] Liu TC , Naito T , Liu Z , Vandussen KL , Haritunians T , Li D , Endo K , Kawai Y , Nagasaki M , Kinouchi Y *et al* (2017b) LRRK2 but not ATG16L1 is associated with Paneth cell defect in Japanese Crohn's disease patients. JCI Insight 2: e91917 28352666 10.1172/jci.insight.91917 PMC5358495

[emmm202216427-bib-0086] Liu TC , Kern JT , VanDussen KL , Xiong S , Kaiko GE , Wilen CB , Rajala MW , Caruso R , Holtzman MJ , Gao F *et al* (2018) Interaction between smoking and ATG16L1T300A triggers Paneth cell defects in Crohn's disease. J Clin Invest 128: 5110–5122 30137026 10.1172/JCI120453 PMC6205411

[emmm202216427-bib-0087] Liu T‐C , Kern JT , Jain U , Sonnek NM , Xiong S , Simpson KF , VanDussen KL , Winkler ES , Haritunians T , Malique A *et al* (2021) Western diet induces Paneth cell defects through microbiome alterations and farnesoid X receptor and type I interferon activation. Cell Host Microbe 29: 988–1001.e6 34010595 10.1016/j.chom.2021.04.004 PMC8192497

[emmm202216427-bib-0088] Lueschow SR , McElroy SJ (2020) The Paneth cell: the curator and defender of the immature small intestine. Front Immunol 11: 587 32308658 10.3389/fimmu.2020.00587 PMC7145889

[emmm202216427-bib-0089] Lueschow SR , Stumphy J , Gong H , Kern SL , Elgin TG , Underwood MA , Kalanetra KM , Mills DA , Wong MH , Meyerholz DK *et al* (2018) Loss of murine Paneth cell function alters the immature intestinal microbiome and mimics changes seen in neonatal necrotizing enterocolitis. PLoS One 13: e0204967 30273395 10.1371/journal.pone.0204967 PMC6166990

[emmm202216427-bib-0090] Małaczewska J , Kaczorek‐ŁUkowska E , Wójcik R , Krzysztof Siwicki A (2019) Antiviral effects of nisin, lysozyme, lactoferrin and their mixtures against bovine viral diarrhoea virus. BMC Vet Res 15: 318 31488163 10.1186/s12917-019-2067-6 PMC6727482

[emmm202216427-bib-0091] Malijauskaite S , Connolly S , Newport D , McGourty K (2021) Gradients in the in vivo intestinal stem cell compartment and their in vitro recapitulation in mimetic platforms. Cytokine Growth Factor Rev 60: 76–88 33858768 10.1016/j.cytogfr.2021.03.002

[emmm202216427-bib-0092] Mastroianni JR , Ouellette A (2009) Defensins in enteric innate immunity. J Biol Chem 284: 27848–27856 19687006 10.1074/jbc.M109.050773 PMC2788835

[emmm202216427-bib-0093] McCarthy N , Manieri E , Storm EE , Saadatpour A , Luoma AM , Kapoor VN , Madha S , Gaynor LT , Cox C , Keerthivasan S *et al* (2020) Distinct mesenchymal cell populations generate the essential intestinal BMP signaling gradient. Cell Stem Cell 26: 391–402.e5 32084389 10.1016/j.stem.2020.01.008 PMC7412576

[emmm202216427-bib-0094] Mei X , Gu M , Li M (2020) Plasticity of Paneth cells and their ability to regulate intestinal stem cells. Stem Cell Res Ther 11: 349 32787930 10.1186/s13287-020-01857-7 PMC7425583

[emmm202216427-bib-0095] Menendez A , Willing BP , Montero M , Wlodarska M , So CC , Bhinder G , Vallance BA , Finlay BB (2013) Bacterial stimulation of the TLR‐MyD88 pathway modulates the homeostatic expression of ileal paneth cell α‐defensins. J Innate Immun 5: 39–49 22986642 10.1159/000341630 PMC6741583

[emmm202216427-bib-0096] Meunier N , O'Connor JM , Maiani G , Cashman KD , Secker DL , Ferry M , Roussel AM , Coudray C (2005) Importance of zinc in the elderly: the ZENITH study. Eur J Clin Nutr 59: S1–S4 10.1038/sj.ejcn.1602286 16254574

[emmm202216427-bib-0097] Meyer‐Hoffert U , Hornef MW , Henriques‐Normark B , Axelsson LG , Midtvedt T , Pütsep K , Andersson M (2008) Secreted enteric antimicrobial activity localises to the mucus surface layer. Gut 57: 764–771 18250125 10.1136/gut.2007.141481

[emmm202216427-bib-0098] Mihaylova MM , Cheng CW , Cao AQ , Tripathi S , Mana MD , Bauer‐Rowe KE , Abu‐Remaileh M , Clavain L , Erdemir A , Lewis CA *et al* (2018) Fasting activates fatty acid oxidation to enhance intestinal stem cell function during homeostasis and aging. Cell Stem Cell 22: 769–778.e4 29727683 10.1016/j.stem.2018.04.001 PMC5940005

[emmm202216427-bib-0099] Mocchegiani E , Giacconi R , Costarelli L (2011) Metalloproteases/anti‐metalloproteases imbalance in chronic obstructive pulmonary disease: genetic factors and treatment implications. Curr Opin Pulm Med 17: S11–S19 22209925 10.1097/01.mcp.0000410743.98087.12

[emmm202216427-bib-0100] Moorefield EC , Andres SF , Blue RE , Van Landeghem L , Amanda T , Santoro MA , Ding S (2017) Aging effects on intestinal homeostasis associated with expansion and dysfunction of intestinal epithelial stem cells. Aging 9: 1898–1915 28854151 10.18632/aging.101279 PMC5611984

[emmm202216427-bib-0101] Mühl H , Bachmann M (2019) IL‐18/IL‐18BP and IL‐22/IL‐22BP: two interrelated couples with therapeutic potential. Cell Signal 63: 109388 31401146 10.1016/j.cellsig.2019.109388

[emmm202216427-bib-0102] Mukherjee P , Chattopadhyay A , Grijalva V , Dorreh N , Lagishetty V , Reddy ST , Fogelman AM (2022) Oxidized phospholipids cause changes in jejunum mucus that induce dysbiosis and systemic in fl ammation. J Lipid Res 63: 100153 34808192 10.1016/j.jlr.2021.100153 PMC8953663

[emmm202216427-bib-0103] Munakata K , Yamamoto M , Anjiki N , Nishiyama M , Imamura S , Iizuka S , Takashima K , Ishige A , Hioki K , Ohnishi Y *et al* (2008) Importance of the interferon‐α system in murine large intestine indicated by microarray analysis of commensal bacteria‐induced immunological changes. BMC Genomics 9: 192 18439305 10.1186/1471-2164-9-192 PMC2408602

[emmm202216427-bib-0104] Murano T , Najibi M , Paulus GLC , Adiliaghdam F , Valencia‐Guerrero A , Selig M , Wang X , Jeffrey K , Xavier RJ , Lassen KG *et al* (2017) Transcription factor TFEB cell‐autonomously modulates susceptibility to intestinal epithelial cell injury *in vivo* . Sci Rep 7: 13938 29066772 10.1038/s41598-017-14370-4 PMC5655326

[emmm202216427-bib-0105] Myer MS (1982) The presence of Paneth cells confirmed in the pig. Onderstepoort J Vet Res 49: 131–132 6184660

[emmm202216427-bib-0106] Nakamura K , Yokoi Y , Fukaya R , Ohira S , Shinozaki R , Nishida T , Kikuchi M , Ayabe T (2020) Expression and localization of Paneth cells and their α‐defensins in the small intestine of adult mouse. Front Immunol 11: 570296 33154750 10.3389/fimmu.2020.570296 PMC7590646

[emmm202216427-bib-0107] Nakanishi Y , Reina‐Campos M , Nakanishi N , Llado V , Elmen L , Peterson S , Campos A , De SK , Leitges M , Ikeuchi H *et al* (2016) Control of Paneth cell fate, intestinal inflammation, and tumorigenesis by PKCλ/ι. Cell Rep 16: 3297–3310 27653691 10.1016/j.celrep.2016.08.054 PMC5043519

[emmm202216427-bib-0108] Nalapareddy K , Helmrath MA , Zheng Y , Nalapareddy K , Nattamai KJ , Kumar RS , Karns R (2017) Canonical wnt signaling ameliorates aging of intestinal stem cells. Cell Rep 18: 2608–2621 28297666 10.1016/j.celrep.2017.02.056 PMC5987258

[emmm202216427-bib-0109] Nalapareddy K , Zheng Y , Geiger H (2022) Aging of intestinal stem cells. Stem Cell Reports 17: 734–740 35276089 10.1016/j.stemcr.2022.02.003 PMC9023768

[emmm202216427-bib-0110] Nandan MO , Ghaleb AM , Liu Y , Bialkowska AB , McConnell BB , Shroyer KR , Robine S , Yang VW (2014) Inducible intestine‐specific deletion of Krüppel‐like factor 5 is characterized by a regenerative response in adult mouse colon. Dev Biol 387: 191–202 24440658 10.1016/j.ydbio.2014.01.002 PMC3949508

[emmm202216427-bib-0111] Nyström EEL , Martinez‐Abad B , Arike L , Birchenough GMH , Nonnecke EB , Castillo PA , Svensson F , Bevins CL , Hansson GC , Johansson MEV (2021) An intercrypt subpopulation of goblet cells is essential for colonic mucus barrier function. Science 372: eabb1590 33859001 10.1126/science.abb1590 PMC8542866

[emmm202216427-bib-0112] Ozcan O , Irmak MK , Dalcik H , Karaoz E , Kubar A , Koylu H (1996) Ultrastructural changes in rat Paneth and goblet cells after the administration of interferon‐alpha. Acta Physiol Hung 84: 81–88 8993678

[emmm202216427-bib-0113] Park SW , Kim M , Brown KM , D'Agati VD , Lee HT (2011) Paneth cell‐derived interleukin‐17A causes multiorgan dysfunction after hepatic ischemia and reperfusion injury. Hepatology 53: 1662–1675 21360570 10.1002/hep.24253 PMC3082595

[emmm202216427-bib-0114] Park SW , Kim M , Kim JY , Ham A , Brown KM , Mori‐Akiyama Y , Ouellette AJ , D'Agati VD , Lee HT (2012) Paneth cell–mediated multiorgan dysfunction after acute kidney injury. J Immunol 189: 5421–5433 23109723 10.4049/jimmunol.1200581 PMC3504173

[emmm202216427-bib-0115] Pasparakis M , Vandenabeele P (2015) Necroptosis and its role in inflammation. Nature 517: 311–320 25592536 10.1038/nature14191

[emmm202216427-bib-0116] Patil A , Hughes AL , Zhang G (2005) Rapid evolution and diversification of mammalian α‐defensins as revealed by comparative analysis of rodent and primate genes. Physiol Genomics 20: 1–11 10.1152/physiolgenomics.00150.2004 15494476

[emmm202216427-bib-0117] Pêgo B , Martinusso CA , Bernardazzi C , Ribeiro BE , De Araujo Cunha AF , De Souza MJ , Nanini HF , Machado MP , Castelo‐Branco MTL , Cavalcanti MG *et al* (2019) Schistosoma mansoni coinfection attenuates murine *Toxoplasma gondii*‐induced Crohn's‐like ileitis by preserving the epithelial barrier and downregulating the inflammatory response. Front Immunol 10: 442 30936867 10.3389/fimmu.2019.00442 PMC6432985

[emmm202216427-bib-0118] Pentinmikko N , Katajisto P (2020) The role of stem cell niche in intestinal aging. Mech Ageing Dev 191: 111330 32805262 10.1016/j.mad.2020.111330

[emmm202216427-bib-0119] Pentinmikko N , Iqbal S , Mana M , Andersson S , Cognetta AB , Suciu RM , Roper J , Luopajärvi K , Markelin E , Gopalakrishnan S *et al* (2019) Notum produced by Paneth cells attenuates regeneration of aged intestinal epithelium. Nature 571: 398–402 31292548 10.1038/s41586-019-1383-0 PMC8151802

[emmm202216427-bib-0120] Perminow G , Beisner J , Koslowski M , Lyckander LG , Stange E , Vatn MH , Wehkamp J (2010) Defective Paneth cell‐mediated host defense in pediatric ileal Crohn's disease. Off J Am Coll Gastroenterol 105: 452–459 10.1038/ajg.2009.643 19904243

[emmm202216427-bib-0121] Podany AB , Wright J , Lamendella R , Soybel DI , Kelleher SL (2016) ZnT2‐mediated zinc import into paneth cell granules is necessary for coordinated secretion and paneth cell function in mice. Cell Mol Gastroenterol Hepatol 2: 369–383 28174721 10.1016/j.jcmgh.2015.12.006 PMC5042355

[emmm202216427-bib-0122] Puiman PJ , Burger‐van Paassen N , Schaart MW , de Bruijn ACJM , de Krijger RR , Tibboel D , van Goudoever JB , Renes IB (2011) Paneth cell hyperplasia and metaplasia in necrotizing enterocolitis. Pediatr Res 69: 217–223 21372757 10.1203/PDR.0b013e3182092a9a

[emmm202216427-bib-0123] Puimège L , Libert C , Van Hauwermeiren F (2014) Regulation and dysregulation of tumor necrosis factor receptor‐1. Cytokine Growth Factor Rev 25: 285–300 24746195 10.1016/j.cytogfr.2014.03.004

[emmm202216427-bib-0124] Purohit V , Bode JC , Bode C , Brenner DA , Choudhry MA , Hamilton F , Kang YJ , Keshavarzian A , Rao R , Sartor RB *et al* (2008) Alcohol, intestinal bacterial growth, intestinal permeability to endotoxin, and medical consequences: Summary of a symposium. Alcohol 42: 349–361 18504085 10.1016/j.alcohol.2008.03.131 PMC2614138

[emmm202216427-bib-0125] Raetz M , Hwang SH , Wilhelm CL , Kirkland D , Benson A , Sturge CR , Mirpuri J , Vaishnava S , Hou B , Defranco AL *et al* (2013) Parasite‐induced T H 1 cells and intestinal dysbiosis cooperate in IFN‐γ‐dependent elimination of Paneth cells. Nat Immunol 14: 136–142 23263554 10.1038/ni.2508 PMC3552073

[emmm202216427-bib-0126] Ragland SA , Criss AK (2017) From bacterial killing to immune modulation: recent insights into the functions of lysozyme. PLoS Pathog 13: 1–22 10.1371/journal.ppat.1006512 PMC5608400 28934357

[emmm202216427-bib-0127] Ren W , Yin J , Duan J , Liu G , Zhu X , Chen S , Li T , Wang S , Tang Y , Hardwidge PR (2014) Mouse intestinal innate immune responses altered by enterotoxigenic *Escherichia coli* (ETEC) infection. Microbes Infect 16: 954–961 25267358 10.1016/j.micinf.2014.09.005

[emmm202216427-bib-0128] Ren Z , Peng L , Chen S , Pu Y , Lv H , Wei H , Wan C (2022) *Lactiplantibacillus plantarum* 1201 inhibits intestinal infection of *Salmonella enterica* subsp. *enterica* serovar typhimurium strain ATCC 13311 in mice with high‐fat diet. Foods 11: 85 10.3390/foods11010085 PMC8750823 35010211

[emmm202216427-bib-0129] Rodriguez NRM , Eloi MD , Huynh A , Dominguez T , Lam HC , Carcamo‐molina D , Naser Z , Desharnais R , Salzman NH , Porter E (2012) Expansion of Paneth cell population in response to enteric *Salmonella enterica* serovar Typhimurium infection. Infect Immun 80: 266–275 22006567 10.1128/IAI.05638-11 PMC3255647

[emmm202216427-bib-0130] Rodríguez‐Colman MJ , Schewe M , Meerlo M , Stigter E , Gerrits J , Pras‐Raves M , Sacchetti A , Hornsveld M , Oost KC , Snippert HJ *et al* (2017) Interplay between metabolic identities in the intestinal crypt supports stem cell function. Nature 543: 424–427 28273069 10.1038/nature21673

[emmm202216427-bib-0131] Rogala AR , Schoenborn AA , Fee BE , Cantillana VA , Joyce MJ , Gharaibeh RZ , Roy S , Fodor AA , Balfour Sartor R , Taylor GA *et al* (2018) Environmental factors regulate Paneth cell phenotype and host susceptibility to intestinal inflammation in Irgm1‐deficient mice. Dis Model Mech 11: dmm031070 29361512 10.1242/dmm.031070 PMC5894938

[emmm202216427-bib-0132] Rumio C , Sommariva M , Sfondrini L , Palazzo M , Morelli D , Viganò L , De Cecco L , Tagliabue E , Balsari A (2012) Induction of Paneth cell degranulation by orally administered Toll‐like receptor ligands. J Cell Physiol 227: 1107–1113 21567398 10.1002/jcp.22830

[emmm202216427-bib-0133] Salzman NH , Ghosh D , Huttner KM , Paterson Y , Bevins CL (2003) Protection against enteric salmonellosis in transgenic mice expressing a human intestinal defensin. Nature 422: 522–526 12660734 10.1038/nature01520

[emmm202216427-bib-0134] Sato T , Vries RG , Snippert HJ , Van De Wetering M , Barker N , Stange DE , Van Es JH , Abo A , Kujala P , Peters PJ *et al* (2009) Single Lgr5 stem cells build crypt‐villus structures *in vitro* without a mesenchymal niche. Nature 459: 262–265 19329995 10.1038/nature07935

[emmm202216427-bib-0135] Sato T , Van Es JH , Snippert HJ , Stange DE , Vries RG , Van Den Born M , Barker N , Shroyer NF , Van De Wetering M , Clevers H (2011a) Paneth cells constitute the niche for Lgr5 stem cells in intestinal crypts. Nature 469: 415–418 21113151 10.1038/nature09637 PMC3547360

[emmm202216427-bib-0136] Sato T , Stange DE , Ferrante M , Vries RGJ , Van Es JH , Van Den Brink S , Van Houdt WJ , Pronk A , Van Gorp J , Siersema PD *et al* (2011b) Long‐term expansion of epithelial organoids from human colon, adenoma, adenocarcinoma, and Barrett's epithelium. Gastroenterology 141: 1762–1772 21889923 10.1053/j.gastro.2011.07.050

[emmm202216427-bib-0137] Satoh Y , Ishikawa K , Oomori Y , Yamano M , Ono K (1989) Effects of cholecystokinin and carbamylcholine on paneth cell secretion in mice: a comparison with pancreatic acinar cells. Anat Rec 225: 124–132 2817426 10.1002/ar.1092250207

[emmm202216427-bib-0138] Sava G , Ceschia V , Pacor S (1989) Mechanism of the antineoplastic action of lysozyme: evidence for host mediated effects. Anticancer Res 9: 1175–1180 2817798

[emmm202216427-bib-0139] Sawada M , Takahashi K , Sawada S , Midorikawa O (1991) Selective killing of Paneth cells by intravenous administration of dithizone in rats. Int J Exp Pathol 72: 407–421 1883741 PMC2001955

[emmm202216427-bib-0140] Scanga CA , Aliberti J , Jankovic D , Tilloy F , Bennouna S , Denkers EY , Medzhitov R , Sher A (2002) Cutting edge: MyD88 is required for resistance to toxoplasma gondii infection and regulates parasite‐induced IL‐12 production by dendritic cells. J Immunol 168: 5997–6001 12055206 10.4049/jimmunol.168.12.5997

[emmm202216427-bib-0141] Schmitt M , Schewe M , Sacchetti A , Feijtel D , van de Geer WS , Teeuwssen M , Sleddens HF , Joosten R , van Royen ME , van de Werken HJG *et al* (2018) Paneth cells respond to inflammation and contribute to tissue regeneration by acquiring stem‐like features through SCF/c‐kit signaling. Cell Rep 24: 2312–2328.e7 30157426 10.1016/j.celrep.2018.07.085

[emmm202216427-bib-0142] Schroeder BO , Ehmann D , Precht JC , Castillo PA , Küchler R , Berger J , Schaller M , Stange EF , Wehkamp J (2015) Paneth cell α‐defensin 6 (HD‐6) is an antimicrobial peptide. Mucosal Immunol 8: 661–671 25354318 10.1038/mi.2014.100 PMC4424388

[emmm202216427-bib-0143] Schwarzer R , Jiao H , Wachsmuth L , Tresch A , Pasparakis M (2020) FADD and Caspase‐8 regulate gut homeostasis and inflammation by controlling MLKL‐ and GSDMD‐mediated death of intestinal epithelial cells. Immunity 52: 978–993.e6 32362323 10.1016/j.immuni.2020.04.002

[emmm202216427-bib-0144] Seki E , Tsutsui H , Tsuji NM , Hayashi N , Adachi K , Nakano H , Futatsugi‐Yumikura S , Takeuchi O , Hoshino K , Akira S *et al* (2002) Critical roles of myeloid differentiation factor 88‐dependent proinflammatory cytokine release in early phase clearance of listeria monocytogenes in mice. J Immunol 169: 3863–3868 12244183 10.4049/jimmunol.169.7.3863

[emmm202216427-bib-0145] Selsted ME , Ouellette AJ (1995) Defensins in granules of phagocytic and non‐phagocytic cells. Trends Cell Biol 5: 114–119 14732166 10.1016/s0962-8924(00)88961-8

[emmm202216427-bib-0146] Shanahan MT , Tanabe H , Ouellette AJ (2011) Strain‐specific polymorphisms in paneth cell α‐defensins of C57BL/6 mice and evidence of vestigial myeloid α‐defensin pseudogenes. Infect Immun 79: 459–573 21041494 10.1128/IAI.00996-10 PMC3019906

[emmm202216427-bib-0147] Shankman LS , Fleury ST , Evans WB , Ravichandran KS , Shankman LS , Fleury ST , Evans WB , Penberthy KK , Arandjelovic S (2021) Efferocytosis by Paneth cells within the intestine. Curr Biol 31: 2469–2476 33852873 10.1016/j.cub.2021.03.055 PMC8281366

[emmm202216427-bib-0148] Shimizu Y , Nakamura K , Yoshii A , Yokoi Y , Kikuchi M , Shinozaki R , Nakamura S , Ohira S , Sugimoto R , Ayabe T (2020) Paneth cell α‐defensin misfolding correlates with dysbiosis and ileitis in Crohn's disease model mice. Life Sci Alliance 3: 1–15 10.26508/lsa.201900592 PMC7190275 32345659

[emmm202216427-bib-0149] Singh R , Balasubramanian I , Zhang L , Gao N (2020) Metaplastic Paneth cells in extra‐intestinal mucosal niche indicate a link to microbiome and inflammation. Front Physiol 11: 280 32296343 10.3389/fphys.2020.00280 PMC7138011

[emmm202216427-bib-0150] Souffriau J , Timmermans S , Vanderhaeghen T , Wallaeys C , Van Looveren K , Aelbrecht L , Dewaele S , Vandewalle J , Goossens E , Verbanck S *et al* (2020) Zinc inhibits lethal inflammatory shock by preventing microbe‐induced interferon signature in intestinal epithelium. EMBO Mol Med 12: 1–21 10.15252/emmm.201911917 PMC7539219 32914580

[emmm202216427-bib-0151] Stappenbeck TS , McGovern DPB (2017) Paneth cell alterations in the development and phenotype of Crohn's disease. Gastroenterology 152: 322–326 27729212 10.1053/j.gastro.2016.10.003 PMC5209278

[emmm202216427-bib-0152] Stockinger S , Albers T , Duerr CU , Ménard S , Pütsep K , Andersson M , Hornef MW (2014) Interleukin‐13‐mediated paneth cell degranulation and antimicrobial peptide release. J Innate Immun 6: 530–541 24556597 10.1159/000357644 PMC6741497

[emmm202216427-bib-0153] Stolzer I , Ruder B , Neurath MF , Günther C (2021) Interferons at the crossroad of cell death pathways during gastrointestinal inflammation and infection. Int J Med Microbiol 311: 151491 33662871 10.1016/j.ijmm.2021.151491

[emmm202216427-bib-0154] Strigli A , Gopalakrishnan S , Zeissig Y , Basic M , Wang J , Schwerd T , Doms S , Peuker K , Hartwig J , Harder J *et al* (2021) Deficiency in X‐linked inhibitor of apoptosis protein promotes susceptibility to microbial triggers of intestinal inflammation. Sci Immunol 6: eabf7473 34739342 10.1126/sciimmunol.abf7473

[emmm202216427-bib-0155] Su D , Nie Y , Zhu A , Chen Z , Wu P , Zhang L , Luo M , Sun Q , Cai L , Lai Y *et al* (2016) Vitamin D signaling through induction of paneth cell defensins maintains gut microbiota and improves metabolic disorders and hepatic steatosis in animal models. Front Physiol 7: 1–18 27895587 10.3389/fphys.2016.00498 PMC5108805

[emmm202216427-bib-0156] Takahashi N , Vanlaere I , De Rycke R , Cauwels A , Joosten LAB , Lubberts E , Van Den Berg WB , Libert C (2008) IL‐17 produced by Paneth cells drives TNF‐induced shock. J Exp Med 205: 1755–1761 18663129 10.1084/jem.20080588 PMC2525583

[emmm202216427-bib-0157] Takashima S , Kadowaki M , Aoyama K , Koyama M , Oshima T , Tomizuka K , Akashi K (2011) The Wnt agonist R‐spondin1 regulates systemic graft‐versus‐host disease by protecting intestinal stem cells. J Exp Med 208: 285–294 21282378 10.1084/jem.20101559 PMC3039850

[emmm202216427-bib-0158] Takashima S , Martin ML , Jansen SA , Fu Y , Bos J , Chandra D , O'Connor MH , Mertelsmann AM , Vinci P , Kuttiyara J *et al* (2019) T cell‐derived interferon‐γ programs stem cell death in immune‐mediated intestinal damage. Sci Immunol 4: eaay8556 31811055 10.1126/sciimmunol.aay8556 PMC7239329

[emmm202216427-bib-0159] Tan G , Zeng B , Zhi FC (2015) Regulation of human enteric α‐defensins by NOD2 in the Paneth cell lineage. Eur J Cell Biol 94: 60–66 25433720 10.1016/j.ejcb.2014.10.007

[emmm202216427-bib-0160] Tanabe H , Ayabe T , Maemoto A , Ishikawa C , Inaba Y , Sato R , Moriichi K , Okamoto K , Watari J , Kono T *et al* (2007) Denatured human α‐defensin attenuates the bactericidal activity and the stability against enzymatic digestion. Biochem Biophys Res Commun 358: 349–355 17482139 10.1016/j.bbrc.2007.04.132

[emmm202216427-bib-0161] Tanaka M , Saito H , Kusumi T (2001) Spatial distribution and histogenesis of colorectal Paneth cell metaplasia in idiopathic inflammatory bowel disease. J Gastroenterol Hepatol 16: 1353–1359 11851832 10.1046/j.1440-1746.2001.02629.x

[emmm202216427-bib-0162] Teltschik Z , Wiest R , Beisner J , Nuding S , Hofmann C , Schoelmerich J , Bevins CL , Stange EF , Wehkamp J (2012) Intestinal bacterial translocation in rats with cirrhosis is related to compromised paneth cell antimicrobial host defense. Hepatology 55: 1154–1163 22095436 10.1002/hep.24789

[emmm202216427-bib-0163] Treveil A , Sudhakar P , Matthews ZJ , Wrzesiński T , Jones EJ , Brooks J , Ölbei M , Hautefort I , Hall LJ , Carding SR *et al* (2020) Regulatory network analysis of Paneth cell and goblet cell enriched gut organoids using transcriptomics approaches. Mol Omi 16: 39–58 10.1039/c9mo00130a 31819932

[emmm202216427-bib-0164] Tschurtschenthaler M , Wang J , Fricke C , Fritz TMJ , Niederreiter L , Adolph TE , Sarcevic E , Künzel S , Offner FA , Kalinke U *et al* (2014) Type I interferon signalling in the intestinal epithelium affects Paneth cells, microbial ecology and epithelial regeneration. Gut 63: 1921–1931 24555997 10.1136/gutjnl-2013-305863

[emmm202216427-bib-0165] Tschurtschenthaler M , Adolph TE , Ashcroft JW , Niederreiter L , Bharti R , Saveljeva S , Bhattacharyya J , Flak MB , Shih DQ , Fuhler GM *et al* (2017) Defective ATG16L1‐mediated removal of IRE1 α drives Crohn's disease‐like ileitis. J Exp Med 214: 401–422 28082357 10.1084/jem.20160791 PMC5294857

[emmm202216427-bib-0166] Vaishnava S , Behrendt CL , Ismail AS , Eckmann L , Hooper LV (2008) Paneth cells directly sense gut commensals and maintain homeostasis at the intestinal host‐microbial interface. Proc Natl Acad Sci U S A 105: 20858–20863 19075245 10.1073/pnas.0808723105 PMC2603261

[emmm202216427-bib-0167] Van Der Flier LG , Clevers H (2009) Stem cells, self‐renewal, and differentiation in the intestinal epithelium. Annu Rev Physiol 71: 241–260 18808327 10.1146/annurev.physiol.010908.163145

[emmm202216427-bib-0168] Van Es JH , Wiebrands K , López‐Iglesias C , Van De Wetering M , Zeinstra L , Van Den Born M , Korving J , Sasaki N , Peters PJ , Van Oudenaarden A *et al* (2019) Enteroendocrine and tuft cells support Lgr5 stem cells on Paneth cell depletion. Proc Natl Acad Sci U S A 116: 26599–26605 31843916 10.1073/pnas.1801888117 PMC6936398

[emmm202216427-bib-0169] Van Hauwermeiren F , Vandenbroucke RE , Grine L , Lodens S , Van Wonterghem E , De Rycke R , De Geest N , Hassan B , Libert C (2015) TNFR1‐induced lethal inflammation is mediated by goblet and Paneth cell dysfunction. Mucosal Immunol 8: 828–840 25425265 10.1038/mi.2014.112

[emmm202216427-bib-0170] Van Looveren K , Libert C (2018) Should we target TNF receptors in the intestinal epithelium with glucocorticoids during systemic inflammation? Expert Opin Ther Targets 22: 1029–1037 30343600 10.1080/14728222.2018.1539078

[emmm202216427-bib-0171] Van Looveren K , Timmermans S , Vanderhaeghen T , Wallaeys C , Ballegeer M , Souffriau J , Eggermont M , Vandewalle J , Van Wyngene L , De Bosscher K *et al* (2020) Glucocorticoids limit lipopolysaccharide‐induced lethal inflammation by a double control system. EMBO Rep 21: e49762 32383538 10.15252/embr.201949762 PMC7332980

[emmm202216427-bib-0172] Vereecke L , Vieira‐Silva S , Billiet T , Van Es JH , Mc Guire C , Slowicka K , Sze M , Van Den Born M , De Hertogh G , Clevers H *et al* (2014) A20 controls intestinal homeostasis through cell‐specific activities. Nat Commun 5: 1–12 10.1038/ncomms6103 25267258

[emmm202216427-bib-0173] Vlantis K , Wullaert A , Polykratis A , Kondylis V , Dannappel M , Schwarzer R , Welz P , Corona T , Walczak H , Weih F *et al* (2016) NEMO prevents RIP Kinase 1‐mediated epithelial cell death and chronic intestinal inflammation by NF‐κB‐dependent and ‐independent functions. Immunity 44: 553–567 26982364 10.1016/j.immuni.2016.02.020 PMC4803910

[emmm202216427-bib-0174] Wahida A , Müller M , Hiergeist A , Popper B , Steiger K , Branca C , Tschurtschenthaler M , Engleitner T , Donakonda S , de Coninck J *et al* (2021) XIAP restrains TNF‐driven intestinal inflammation and dysbiosis by promoting innate immune responses of Paneth and dendritic cells. Sci Immunol 6: eabf7235 34739338 10.1126/sciimmunol.abf7235

[emmm202216427-bib-0175] Wang H , Zhang X , Zuo Z , Zhang Q , Pan Y , Zeng B , Li W , Wei H , Liu Z (2017a) Rip2 is required for Nod2‐mediated lysozyme sorting in Paneth cells. J Immunol 198: 3729–3736 28330897 10.4049/jimmunol.1601583

[emmm202216427-bib-0176] Wang Q , Zhou Y , Rychahou P , Fan TWM , Lane AN , Weiss HL , Evers BM (2017b) Ketogenesis contributes to intestinal cell differentiation. Cell Death Differ 24: 458–468 27935584 10.1038/cdd.2016.142 PMC5344206

[emmm202216427-bib-0177] Wang SL , Shao BZ , Zhao SB , Fang J , Gu L , Miao CY , Li ZS , Bai Y (2018) Impact of paneth cell autophagy on inflammatory bowel disease. Front Immunol 9: 693 29675025 10.3389/fimmu.2018.00693 PMC5895641

[emmm202216427-bib-0178] Wang Y , Song W , Wang J , Wang T , Xiong X , Qi Z , Fu W , Yang X , Chen YG , Chen YG (2020) Single‐cell transcriptome analysis reveals differential nutrient absorption functions in human intestine. J Exp Med 217: e20191130 31753849 10.1084/jem.20191130 PMC7041720

[emmm202216427-bib-0179] Wehkamp J , Stange EF (2020) An update review on the Paneth cell as key to ileal Crohn's disease. Front Immunol 11: 646 32351509 10.3389/fimmu.2020.00646 PMC7174711

[emmm202216427-bib-0180] Wehkamp J , Wang G , Kübler I , Nuding S , Gregorieff A , Schnabel A , Kays RJ , Fellermann K , Burk O , Schwab M *et al* (2007) The Paneth cell α‐defensin deficiency of ileal Crohn's disease is linked to Wnt/Tcf‐4. J Immunol 179: 3109–3118 17709525 10.4049/jimmunol.179.5.3109

[emmm202216427-bib-0181] Welz PS , Wullaert A , Vlantis K , Kondylis V , Fernández‐Majada V , Ermolaeva M , Kirsch P , Sterner‐Kock A , Van Loo G , Pasparakis M (2011) FADD prevents RIP3‐mediated epithelial cell necrosis and chronic intestinal inflammation. Nature 477: 330–334 21804564 10.1038/nature10273

[emmm202216427-bib-0182] White JR , Gong H , Pope B , Schlievert P , Mcelroy SJ (2017) Paneth‐cell‐disruption‐induced necrotizing enterocolitis in mice requires live bacteria and occurs independently of TLR4 signaling. Dis Model Mech 10: 727–736 28450472 10.1242/dmm.028589 PMC5483007

[emmm202216427-bib-0183] Wielockx B , Libert C , Wilson C (2004) Matrilysin (matrix metalloproteinase‐7): a new promising drug target in cancer and inflammation? Cytokine Growth Factor Rev 15: 111–115 15110795 10.1016/j.cytogfr.2003.12.001

[emmm202216427-bib-0184] Wilson CL , Ouellette AJ , Satchell DP , Ayabe T , López‐Boado YS , Stratman JL , Hultgren SJ , Matrisian LM , Parks WC (1999) Regulation of intestinal α‐defensin activation by the metalloproteinase matrilysin in innate host defense. Science 286: 113–117 10506557 10.1126/science.286.5437.113

[emmm202216427-bib-0185] Wittkopf N , Günther C , Martini E , Waldner M , Amann KU , Neurath MF , Becker C (2012) Lack of intestinal epithelial Atg7 affects paneth cell granule formation but does not compromise immune homeostasis in the gut. Clin Dev Immunol 2012: 278059 22291845 10.1155/2012/278059 PMC3265132

[emmm202216427-bib-0186] Wu A , Yu B , Zhang K , Xu Z , Wu D , He J , Luo J , Luo Y , Yu J , Zheng P *et al* (2020) Transmissible gastroenteritis virus targets Paneth cells to inhibit the self‐renewal and differentiation of Lgr5 intestinal stem cells via Notch signaling. Cell Death Dis 11: 40 31959773 10.1038/s41419-020-2233-6 PMC6971083

[emmm202216427-bib-0187] Yang Q , Bermingham NA , Finegold MJ , Zoghbi HY (2001) Requirement of Math1 for secretory cell lineage commitment in the mouse intestine. Science 294: 2155–2158 11739954 10.1126/science.1065718

[emmm202216427-bib-0188] Yilmaz ÖH , Katajisto P , Lamming DW , Gültekin Y , Bauer‐Rowe KE , Sengupta S , Birsoy K , Dursun A , Onur Yilmaz V , Selig M *et al* (2012) MTORC1 in the Paneth cell niche couples intestinal stem‐cell function to calorie intake. Nature 486: 490–495 22722868 10.1038/nature11163 PMC3387287

[emmm202216427-bib-0189] Yokoi Y , Adachi T , Sugimoto R , Kikuchi M , Ayabe T , Nakamura K (2021) Simultaneous real‐time analysis of Paneth cell and intestinal stem cell response to interferon‐γ by a novel stem cell niche tracking method. Biochem Biophys Res Commun 545: 14–19 33529805 10.1016/j.bbrc.2021.01.050

[emmm202216427-bib-0190] Yu S , Tong K , Zhao Y , Balasubramanian I , Yap GS , Ferraris RP , Bonder EM , Verzi MP , Gao N (2018) Paneth cell multipotency induced by notch activation following injury. Cell Stem Cell 23: 46–59.e5 29887318 10.1016/j.stem.2018.05.002 PMC6035085

[emmm202216427-bib-0191] Zaragoza MM , Sankaran‐Walters S , Canfield DR , Hung JKS , Martinez E , Ouellette AJ , Dandekar S (2011) Persistence of gut mucosal innate immune defenses by enteric α‐defensin expression in the simian immunodeficiency virus model of AIDS. J Immunol 186: 1589–1597 21178012 10.4049/jimmunol.1002021 PMC4052980

[emmm202216427-bib-0192] Zhang Y , Cougnon FBL , Wanniarachchi YA , Hayden JA , Nolan EM (2013) Reduction of human defensin 5 affords a high‐affinity zinc‐chelating peptide. ACS Chem Biol 8: 1907–1911 23841778 10.1021/cb400340k PMC3783636

[emmm202216427-bib-0193] Zhang Q , Pan Y , Yan R , Zeng B , Wang H , Zhang X , Li W , Wei H , Liu Z (2015) Commensal bacteria direct selective cargo sorting to promote symbiosis. Nat Immunol 16: 918–926 26237551 10.1038/ni.3233

[emmm202216427-bib-0194] Zhao D , Kim Y , Jeong S , Greenson JK , Chaudhry MS , Hoepting M , Anderson ER , Van Den BMRM , Peled JU , Gomes ALC *et al* (2018) Survival signal REG3 α prevents crypt apoptosis to control acute gastrointestinal graft‐versus‐host disease. J Clin Invest 128: 3–12 10.1172/JCI99261 PMC6205404 30106382

[emmm202216427-bib-0195] Zhong W , Wei X , Hao L , Du LT , Yue R , Sun X , Guo W , Dong H , Li T , Ahmadi AR *et al* (2020) Paneth cell dysfunction mediates alcohol‐related steatohepatitis through promoting bacterial translocation in mice: role of zinc deficiency. Hepatology 71: 1575–1591 31520476 10.1002/hep.30945 PMC7069794

[emmm202216427-bib-0196] Zwarycz B , Gracz AD , Rivera KR , Williamson IA , Samsa LA , Starmer J , Daniele MA , Salter‐Cid L , Zhao Q , Magness ST (2019) IL22 inhibits epithelial stem cell expansion in an ileal organoid model. Cell Mol Gastroenterol Hepatol 7: 1–17 30364840 10.1016/j.jcmgh.2018.06.008 PMC6199238

